# MAGI1, a New Potential Tumor Suppressor Gene in Estrogen Receptor Positive Breast Cancer

**DOI:** 10.3390/cancers12010223

**Published:** 2020-01-16

**Authors:** Begoña Alday-Parejo, François Richard, Janine Wörthmüller, Tilman Rau, José A. Galván, Christine Desmedt, Albert Santamaria-Martinez, Curzio Rüegg

**Affiliations:** 1 Laboratory of Experimental and Translational Oncology, Pathology, Department of Oncology, Microbiology and Immunology, Faculty of Sciences and Medicine, University of Fribourg, 1700 Fribourg, Switzerland; begona.aldayparejo@unifr.ch (B.A.-P.); janine.woerthmueller@unifr.ch (J.W.); 2 Laboratory for Translational Breast Cancer Research, KU Leuven, 3001 Leuven, Belgium; francois.richard@kuleuven.be; 3 Institute of Pathology, University of Bern, 3008 Bern, Switzerland; tilman.rau@pathology.unibe.ch (T.R.); jose.galvan@pathology.unibe.ch (J.A.G.); 4 Tumor Ecology Laboratory, Pathology, Department of Oncology, Microbiology and Immunology, Faculty of Science and Medicine, University of Fribourg, 1700 Fribourg, Switzerland; albert.santamaria@unifr.ch

**Keywords:** breast cancer, MAGI1, prognosis, stratification, estrogen receptor, recurrence, inflammation, COX-2, metastasis

## Abstract

Membrane-associated guanylate kinase (MAGUK) with inverted domain structure-1 (MAGI1) is an intracellular adaptor protein that stabilizes epithelial junctions consistent with a tumor suppressive function in several cancers of epithelial origin. Here we report, based on experimental results and human breast cancer (BC) patients’ gene expression data, that MAGI1 is highly expressed and acts as tumor suppressor in estrogen receptor (ER)^+^/HER2^−^ but not in HER2^+^ or triple negative breast cancer (TNBC). Within the ER^+^/HER2^−^ subset, high MAGI1 expression associates with ESR1 and luminal genes GATA3 and FOXA1 expression and better prognosis, while low MAGI1 levels correlates with higher histological grade, more aggressive phenotype and worse prognosis. Experimentally, MAGI1 downregulation in the ER^+^ human BC cells MCF7 impairs ER expression and signaling, promotes cell proliferation, and reduces apoptosis and epithelial differentiation. MAGI1 downregulation in the ER^+^ murine BC cell line 67NR accelerates primary tumor growth and enhances experimental lung metastasis formation. MAGI1 expression is upregulated by estrogen/ER, downregulated by prostaglandin E2/COX-2axis, and negatively correlates with inflammation in ER^+^/HER2^−^ BC patients. Taken together, we show that MAGI1 is a new potential tumor suppressor in ER^+^/HER2^−^ breast cancer with possible prognostic value for the identification of patients at high-risk of relapse within this subset.

## 1. Introduction

MAGI1 is a cytoplasmic scaffolding protein present at cell-to-cell contacts [1,2,3]. MAGI1 expression is downregulated during cancer progression and was proposed to act as a tumor suppressor in several cancers, including hepatocellular carcinoma, colorectal, cervical and gastric cancers [4,5,6,7]. We have previously shown that silencing MAGI1 expression in colorectal cancer (CRC) cells promotes primary tumor growth and metastasis associated with loss of E-cadherin at cell contacts and Wnt signaling activation, while MAGI1 overexpression had opposite effects. We therefore proposed that MAGI1 is a tumor suppressor in CRC [4]. Furthermore, MAGI1 interacts with the tumor suppressor PTEN leading to PTEN and E-cadherin stability at cell–cell contacts [8,9,10,11]. Stimulated by these observations we sought to analyze whether MAGI1 may also act as a tumor suppressor in breast cancer.

Breast cancer (BC) is a heterogeneous disease that differs at the genetic, histopathological and clinical levels [12]. Three clinically relevant biological BC subtypes have been defined: Estrogen/Progesterone Receptor positive (ER^+^/PR^+^), Human Epidermal growth factor Receptor 2 amplified (HER2^+^) and triple negative (TNBC; i.e., ER^−^/PR-/HER2^−)^ BC [13]. The introduction of anti-estrogen (e.g., tamoxifen) and anti-HER2 (e.g., Herceptin)-based adjuvant treatments for ER^+^ and HER2^+^ BC, respectively, in combination with mastectomy or breast-saving surgery, have improved survival by about 30% in the past three decades [14]. For TNBC there are still no specific established therapies due to lack of defined targets, and radio- and chemotherapies are commonly used instead [15]. Additional potential therapeutic targets for personalized therapies have been identified (e.g., PI3KCA, EGFR, PARP, and PDL/PDL-1) [16,17,18]. Gene expression profiling classified BC into different molecular subtypes with distinct features and clinical outcomes: luminal A, luminal B, HER2-enriched, basal-like, claudin-low and normal-like subtypes [19,20,21,22]. This molecular classification contributed to refine the diagnosis and selection of the most appropriate therapy. For instance, among the estrogen/progesterone receptor positive (ER^+^/PR^+^) tumors expressing luminal epithelial genes and ER target genes, a large subset has low proliferation, responds well to anti-estrogen (hormonal) therapy with ER antagonists, such as tamoxifen or fulvestrant, and has a favorable outcome [23]. This subset is also referred to as luminal A. However, some ER^+^ breast cancers have a higher risk for recurrence and poorer prognosis, especially in younger women and benefit more from adjuvant chemotherapy [24,25,26]. In these patients, tumors are of higher grade, more proliferative, and have a higher risk of metastatic progression [24,25,26,27]. These highly proliferative ER^+^ breast cancers are referred to as luminal B [19,20,21,22]. These tumors have lower expression of luminal and ER target genes, indicating that they may rely upon alternative pathways for growth (e.g., EGFR/HER2/MAPK or PI3K/AKT) [28,29]. In consequence, highly proliferative ER^+^ BC at high risk of recurrence may benefit more from a combined therapeutic strategy including adjuvant chemo- and hormonal therapies [25,26,30]. In this context, tests based on gene-expression in the primary tumor, have shown clinical potential in identifying patients with early BC with an excellent prognosis who could be spared of adjuvant chemotherapy [24,25,26,31,32,33]. It remains nevertheless of critical importance to identify molecular drivers of progression in ER^+^/HER2^−^ BC to further refine therapeutic approach in the high-risk subset of ER^+^/HER2^−^ BC patients.

Here, using a combined experimental and clinical approach, we report the identification of MAGI1 as tumor suppressor in ER^+^/HER2^−^ BC with possible prognostic value for the identification of patients at high-risk of relapse within this subset.

## 2. Results

### 2.1. MAGI1 Is Highly Expressed in ER^+^ Breast Cancer

**Figure 1:**
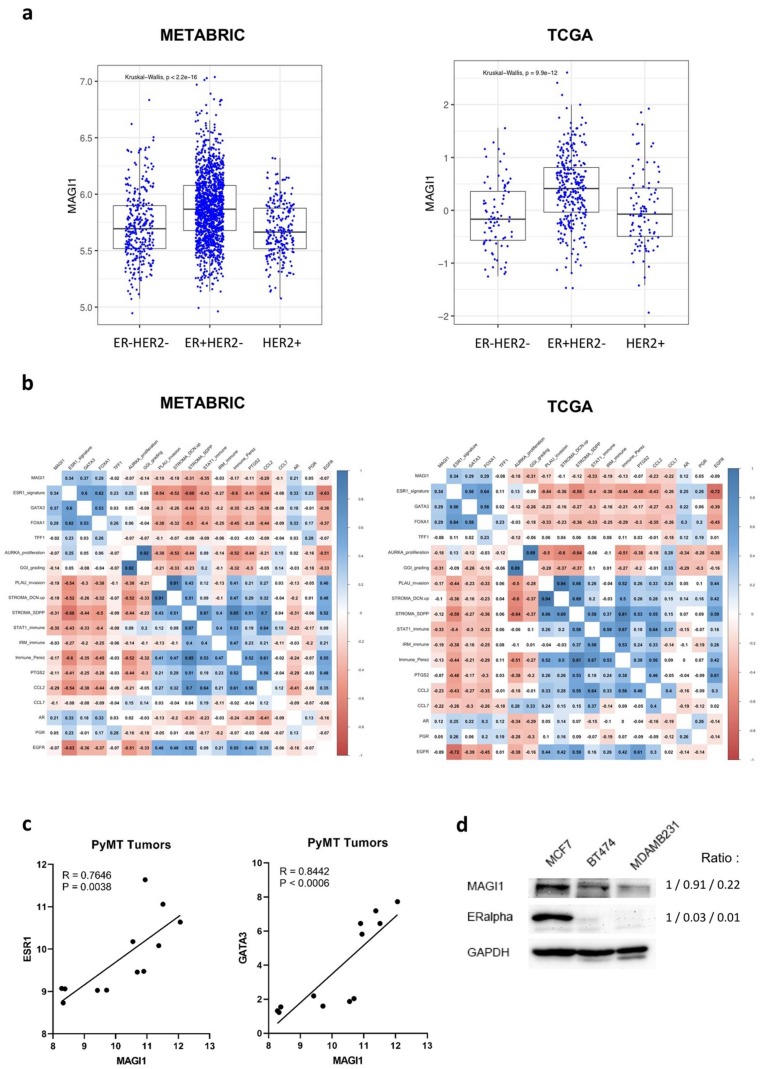
Membrane-associated guanylate kinase with inverted domain structure-1 (MAGI1) expression correlates with estrogen receptor breast cancer (ER+ BC) subtype. (**a**) MAGI1 expression from METABRIC and TCGA microarray analysis by BC subtype ER^−^/HER2^−^, ER^+^/HER2^−^, and HER2^+^. Statistical analysis was performed by Kruskal–Wallis test (non-parametric one-way ANOVA). (**b**) Heat plot correlation matrices in ER^+^HER2^−^ patients from METABRIC and TCGA data. Significant correlations are in blue, anti-correlations are in red. (**c**) MAGI1 mRNA levels positively correlates with ESR1 and GATA3 expression in MMTV-PyMT tumor samples. The data from qPCR are shown as a percentage of *Arbp*1. Correlation analysis was calculated with Pearson’s R correlation test (*n* = 12 tumors). (**d**) Western blot showing MAGI1 and ERα protein levels in MCF7 (ER^+^/HER2^−^), BT474 (HER2^+^), and MDA-MB-231 (basal-like, ER^−^/HER2^−^) cell lines. GAPDH is used as loading control. Band intensity ratio adjusted to GAPDH is shown next to the blot.

MAGI1 expression was shown to be downregulated in several cancers, including hepatocellular carcinoma, colorectal, cervical, and gastric cancers [4,5,6,7], but little is known about its expression in BC and its subtypes. To address this question, we analyzed METABRIC and TCGA gene expression data sets [34,35] of primary human breast cancers and observed that MAGI1 expression is higher in ER^+^/HER2^−^ compared to HER2^+^ and ER^−^/HER2^−^ BC subtypes (Figure 1a). Consistent with this observation, MAGI1 expression positively correlates with ESR1 (the gene coding for ER) and luminal fate transcription factors GATA3 and FOXA1 expression within ER+/HER2^−^ breast cancers in both data sets (Figure 1b, significant correlations are in blue, anti-correlations are in red). A similar positive correlation between MAGI1, ESR1, and GATA3 expression is also found in tumor samples derived from the MMTV-PyMT spontaneous model of BC (Figure 1c). Additionally, and consistently, MAGI1 protein is highly expressed in the ER^+^ human cell line MCF7 compared to HER2^+^ BT-474 cell line and the basal like/TNBC-derived MDA-MB-231 cell line (Figure 1d).

These results indicate that in BC, MAGI1 expression is higher in the ER^+^ subtype and positively correlated with the expression of ESR1 and the luminal genes GATA3 and FOXA1.

### 2.2. MAGI1 Is Upregulated by Estrogen Receptor Alpha (erα) and Contributes to ER Signaling

**Figure 2:**
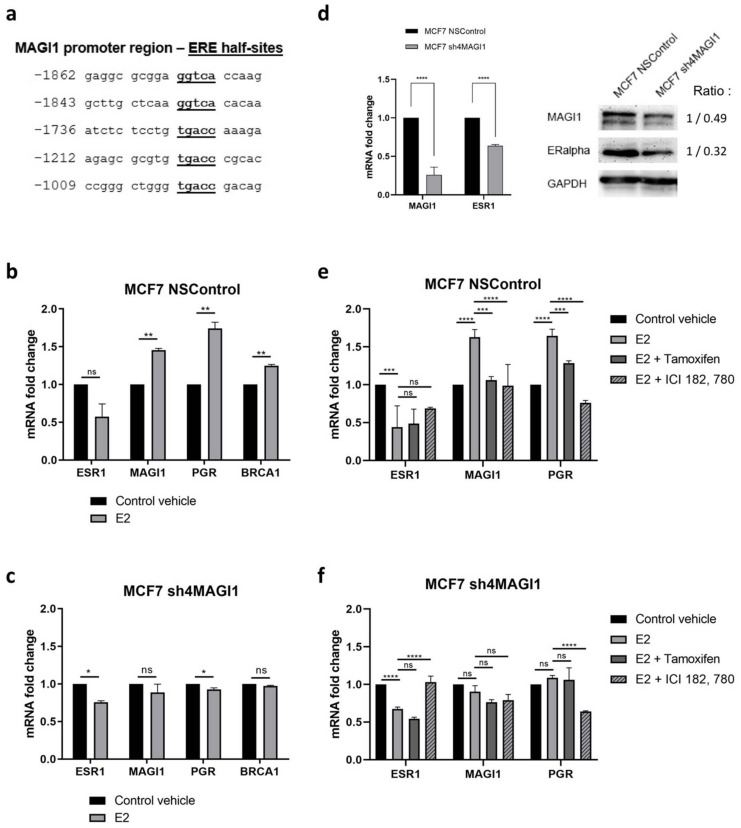
MAGI1 is upregulated by estrogen receptor (ER) α and MAGI1 contributes to ER signaling (**a**). Schematic representation of estrogen response element (ERE) half-site motifs in MAGI1 promoter sequence. The adenine (**a**) of the first codon, atg, is numbered as 1. The sequences of ERE sites I-V core regions are underlined and highlighted in bold. (**b**,**c**) Real time PCR quantification of (**b**) ESR1, MAGI1, PGR, and BRCA1 mRNA in MCF7 NSControl and (**c**) MCF7 sh4MAGI1 upon 17β-estradiol (E2) treatment (*n* = 3 independent experiments, each analyzed in triplicate) (**d**). Western blot and qPCR showing MAGI1 and ERα protein and mRNA levels, respectively, in MCF7 NSControl and MCF7 sh4MAGI1 cells. GAPDH is used as loading control. Band intensity ratio adjusted to GAPDH is shown next to the blot. (**e**,**f**) Real time PCR quantification of ESR1, MAGI1 and PGR mRNA in (**e**) MCF7 NSControl and (**f**) MCF7 sh4MAGI1 upon 17β-estradiol (E2) treatment alone or in the presence of tamoxifen or ICI 182,780 (or vehicle only, control) as indicated (*n* = 3 independent experiments, each analyzed in triplicate). qPCR data are shown as percentage of the value of the housekeeping gene GAPDH, and represent mean values ± S.D. Statistical analysis was performed by unpaired *t*-test. ns = no statistical difference, * *p* < 0.05, ** *p* < 0.01, *** *p* < 0.005, **** *p* < 0.001.

To investigate whether MAGI1 may be regulated by estrogen and ERα, we first analyzed bioinformatically the MAGI1 promoter sequence and noticed that it contains five different putative estrogen response elements (EREs) half-sites at positions −1009/−1013 (Site I), −1212/−1216 (Site II), −1736/−1740 (Site III); −1843/−1847 (Site IV) and −1862/−1866 (Site V) (Figure 2a). To functionally test whether MAGI1 mRNA expression is indeed regulated by estrogen, MCF7 cells were serum-starved for 48 h and subsequently treated for 6 h with 10^−6^ M 17β-estradiol (E2) or vehicle only. As shown in Figure 2b, MAGI1 mRNA levels were up-regulated upon E2 treatment together with progesterone receptor (PGR) and BRCA1, two known estrogen regulated genes [36]. ESR1 expression, which is known to be negatively regulated by E2 itself [37], showed a trend toward reduced expression but the difference was not significant. Next, we tested whether MAGI1 itself functionally contributed to E2/ER signaling. MAGI1 downregulation in MCF7 cells prevented induction of PGR and BRCA1 expression in response to E2 stimulation (Figure 2c). MAGI1 downregulation in MCF7 cells decreased ESR1 mRNA and ERα protein levels (Figure 2d). To gather additional evidence that these effects were E2/ER specific, we stimulated MCF7 control cells (NSControl) and MCF7 cells with downregulated MAGI1 (sh4MAGI1), with E2 in the presence of the ER antagonist tamoxifen and ICI 182,780, and measured expression of MAGI1, ESR1 and PGR by RT-qPCR. Results show that tamoxifen and ICI 182,780 blunted the effects of E2 stimulation on MAGI1, ESR1 and PGR expression (Figure 2e,f). Taken together these data suggests the involvement of MAGI1 on ER signaling.

To complement experimental results, we performed a gene ontology (GO) analysis for biological processes on the above-mentioned human data sets, focusing only on ER^+^/HER2^−^ BC. This analysis revealed that MAGI1 expression positively correlates with biological processes consistent with increased ER activity such as transcription from RNA polymerase II (GO:0006357, q = 1.41 × 10^−13^), gene expression, RNA metabolism, macromolecule biosynthesis, transport and localization and histone modifications (Figure S1a, Table S1).

Taken together these results indicate that MAGI1 is regulated by E2/ERα and at the same time is required for expression of ERα and ERα-dependent genes in ER^+^ BC cells.

### 2.3. MAGI1 Downregulation Increases Proliferation, Reduces Apoptosis and Activates PI3K/Wnt Signaling in MCF7 Cells

**Figure 3:**
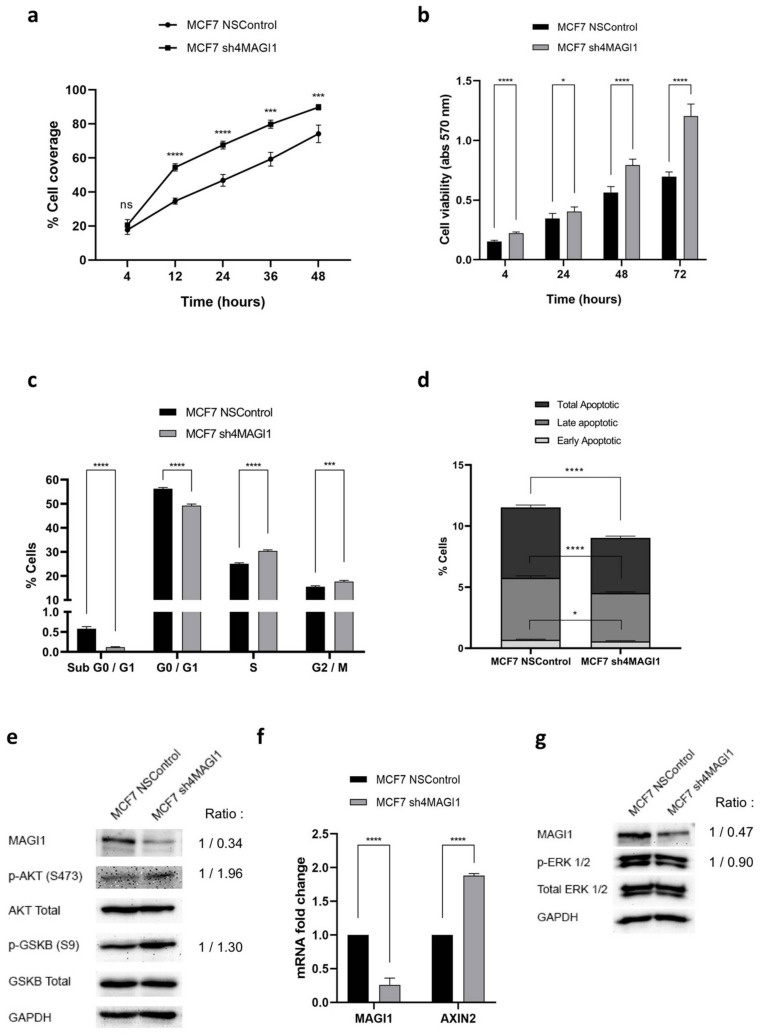
MAGI1 downregulation increases proliferation, reduces apoptosis and activates PI3K/Wnt signaling in MCF7 cells. (**a**) Growth of MCF7 NSControl and MCF7 sh4MAGI1 cells measured as percentage of cell culture well coverage by the IncuCyte system at the indicated time points (hours) after plating (*n* = 18 independent experiments). (**b**) Cell viability of MCF7 NSControl and MCF7 sh4MAGI1 cells measured by MTT assay and the indicated time points (hours) after plating (*n* = 6 independent experiments). (**c**) Quantification of the percentage of MCF7 cells in the sub G0/G1, G0/G1, S and G2/M phases of the cell cycle by Edu/DAPI staining (*n* = 3 independent experiments). (**d**) Quantification of the percentage of early, late and total apoptotic MCF7 cells by DAPI/Annexin-V staining and analysis. The data represents mean values ± S.D. (*n* = 3 independent experiments). (**e**) Effect of MAGI1 silencing (sh4MAGI1) on S473AKT and S9GSKβ3 phosphorylation relative to control (NSControl) in MCF7 cells revealed by western blotting analysis. (**f**) Effect of MAGI1 silencing (sh4MAGI1) relative to control (NSControl) MCF7 cells on AXIN2 expression. The data are shown as percentage of the value of the housekeeping gene GAPDH, and represent mean values ± S.D. (*n* = 3 independent experiments, each analyzed in triplicate). (**g**) Effect of MAGI1 silencing (sh4MAGI1) on ERK phosphorylation relative to control (NSControl) in MCF7 cells revealed by western blotting analysis. GAPDH is used as loading control. Band intensity ratio adjusted to GAPDH is shown next to the blot. Statistical analyses were performed by unpaired *t*-test. ns = no statistical difference, * *p* < 0.05, *** *p* < 0.005, **** *p* < 0.001.

ER is a mitogenic pathway for normal breast epithelial cells and ER^+^ BC cells, however, loss of ER expression/function is associated with increased proliferation and aggressiveness [38]. To test whether MAGI1 modulation may affect cell growth, we monitored cell proliferation, cell cycle progression, survival, and apoptosis in MCF7 cells with silenced MAGI1 compared to non-silenced (NS) control MCF7 cells. MAGI1 silencing (sh4MAGI1) increased MCF7 growth and survival compared to non-silenced (NSControl) cells (Figure 3a,b). Consistently, cell cycle analysis shows that MCF7 cultures with downregulated MAGI1 have an increased number of cells in S phase and a decreased number in G0/G1 phase (Figure 3c). Interestingly, the cell population in sub G0/G1 phase, representing cells with DNA fragmentation, is also reduced in MCF7 cells with downregulated MAGI1, suggesting a possible effect of MAGI1 downregulation in apoptosis. Annexin-V/DAPI staining analysis confirmed that MCF7 with downregulated MAGI1 have reduced apoptosis compared to NSControl cells (Figure 3d). Additionally, in the TCGA dataset, and to a lesser extent in the METABRIC dataset, we observed that MAGI1 expression is negatively correlated with a proliferative signature (AURKA Proliferation) in ER^+^/HER2^−^ BC (See Figure 1b). Consistently, gene ontology analysis in human BC samples revealed that MAGI1 expression negatively correlates with GO terms involved in the regulation of chromosome segregation, cell division and cell proliferation (Figure S1b, Table S2).

Next, we tested whether silencing MAGI1 in cells with low expression levels may also affect proliferation. For this we used the isogenic cell lines 4T1 (ER^−^ expressing low MAGI1 levels) to compare it to 67NR (ER^+^ expressing high MAGI1 levels) cells (Figure S2a). Consistent with what we observed with MCF7 cells, MAGI1 silencing in 67NR cells increased proliferation (Figure S2b). Surprisingly, however, MAGI1 silencing in 4T1 cells also enhanced proliferation (Figure S2c). These results suggested that silencing MAGI1 may render ER^+^ cells (e.g., MCF7 and 67NR) more sensitive to chemotherapy. To this end, we treated non-silenced and MAGI1-silenced MCF7 and 67NR cells with increasing concentrations of methotrexate and doxorubicin, two drugs used in BC adjuvant chemotherapy [39], to determine the IC50 cytotoxic concentrations. Results from this experiment showed that in both cell lines MAGI1 silencing did not increase sensitivity to the tested drugs (Figure S2d,e).

In complement to these experiments we tested the effect of overexpressing MAGI1b isoform on the proliferation of the TNBC cell line MDA-MB-231. Overexpression was confirmed by RT-qPCR and western blotting (Figure S3a,b). MAGI1b overexpression, however, had no effect on MDA-MB-231 proliferation and survival (Figure S3c,d).

As MAGI1 downregulation decreases E2/ER-signaling, it is likely that the increased proliferation observed in MCF7 may involve an alternative pathway. The PI3K-AKT signaling pathway is activated in over 60% of ER^+^ breast cancer and contributes to resistance to ER inhibition and progression [40,41] and MCF7 cells carry mutations typically found in human luminal breast cancers, including PIK3CA E545K [42,43]. Interestingly, MAGI1 downregulation increased phosphorylation (S473) of AKT, a direct target of PI3K, and phosphorylation (S9) of GSKβ3, itself a direct target of AKT [44], and regulator of the Wnt pathway (Figure 3e). Consistent with GSKβ3 inhibition (phosphorylation) we observed increased levels of the Wnt target Axin-2 [45] (Figure 3f). In contrast, MAGI1 downregulation did not alter the phosphorylation of Extracellular Regulated Kinase (ERK) 1/2 (Figure 3g).

We conclude that MAGI1 silencing in MCF7 cells activated the PI3K signaling and Wnt pathways and promoted MCF7proliferation and survival.

### 2.4. MAGI1 Downregulation in MCF7 Breast Cancer Cells Reduces Epithelial Differentiation

**Figure 4:**
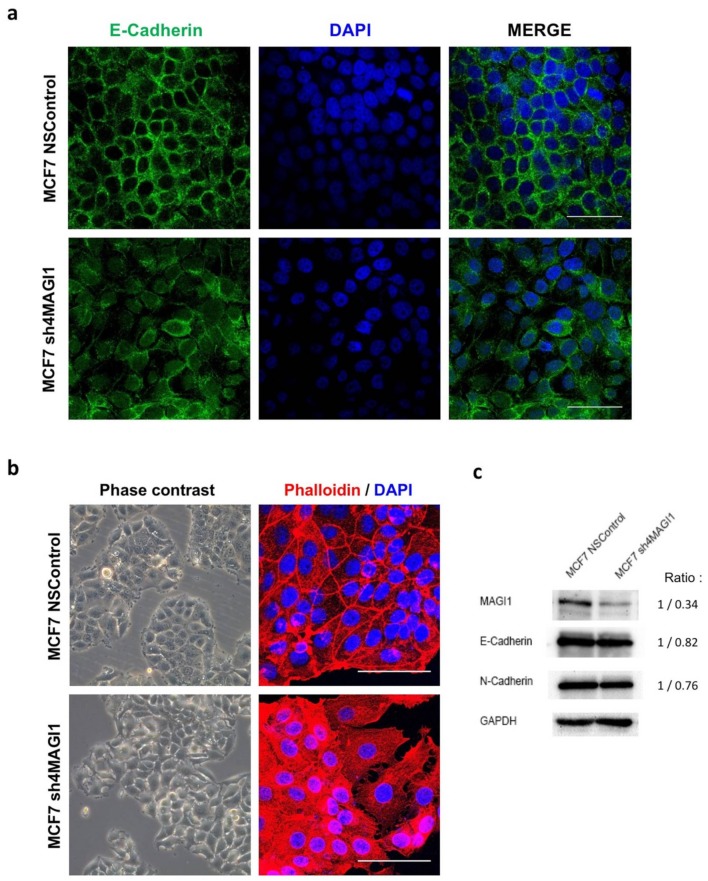
MAGI1 downregulation in MCF7 breast cancer cells reduces epithelial differentiation. (**a**) Confocal images of immunofluorescence staining of DAPI (blue), E-cadherin (green) and merged images in MCF7 NSControl and MCF7 sh4MAGI1 cells (63× objective, 1.5× zoom). (**b**) Representative phase-contrast images (20× objective) and confocal images of immunofluorescence staining of phalloidin (red) and DAPI (blue) (63× objective, 2× zoom) of MCF7 NSControl and MCF7 sh4MAGI1 cells. Scale bars correspond to 50 µm. (**c**) Western blot showing MAGI1, E-cadherin, and N-cadherin protein levels in MCF7 NSControl and MCF7 sh4MAGI1 cells. GAPDH is used as loading control. Band intensity ratio adjusted to GAPDH is shown next to the blot.

MAGI1 downregulation in the BC cell line MCF7 leads to loss of E-cadherin localization at cellular junctions (Figure 4a). In sub-confluent cultures, MCF7 typically forms regular islands of cohesive, polygonal epithelial cells. MAGI1 downregulation altered this phenotype and induced irregular islands with elongated or rounded cells and rearranged actin fibers from subcortical to cytoplasmic localization (Figure 4b). However, MAGI1 downregulation did not induce global reduction of E-cadherin nor increase of N-cadherin expression (Figure 4c), indicating that MAGI1 downregulation does not cause epithelial-to-mesenchymal transition but rather a loss of epithelial differentiation traits.

**Figure 5:**
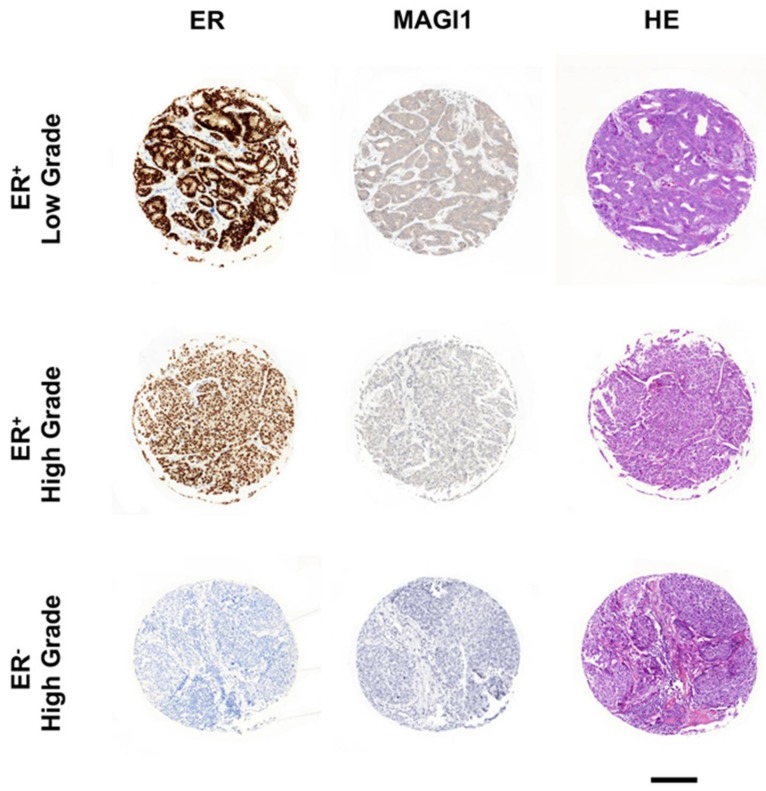
Immunohistochemical analysis of ER and MAGI1 expression in human breast cancers. MAGI1 expression is well expressed in low grade (well differentiated) ER^+^ tumors, expressed in lower level in high grade (poorly differentiated) ER^+^ tumors and absent in ER^−^ tumors. Differentiation (grading) was determined by HE staining. Scale bar corresponds to 200 µm.

These observations indicate that reduced MAGI1 expression favors loss of differentiation of BC cells. In order to corroborate this observation with clinical evidence, we performed HE, ER, and MAGI1 IHC staining of human breast cancer tissues. We observed that MAGI1 is expressed in well differentiated ER^+^ tumors, expressed in lower levels in poorly differentiated ER^+^ and absent in ER^−^ BC (Figure 5). From these results we conclude that MAGI1 expression in BC is associated with and contributes to a differentiated phenotype.

### 2.5. MAGI1 Downregulation Promotes Metastasis of Disseminated ER^+^ Cancer Cells

**Figure 6:**
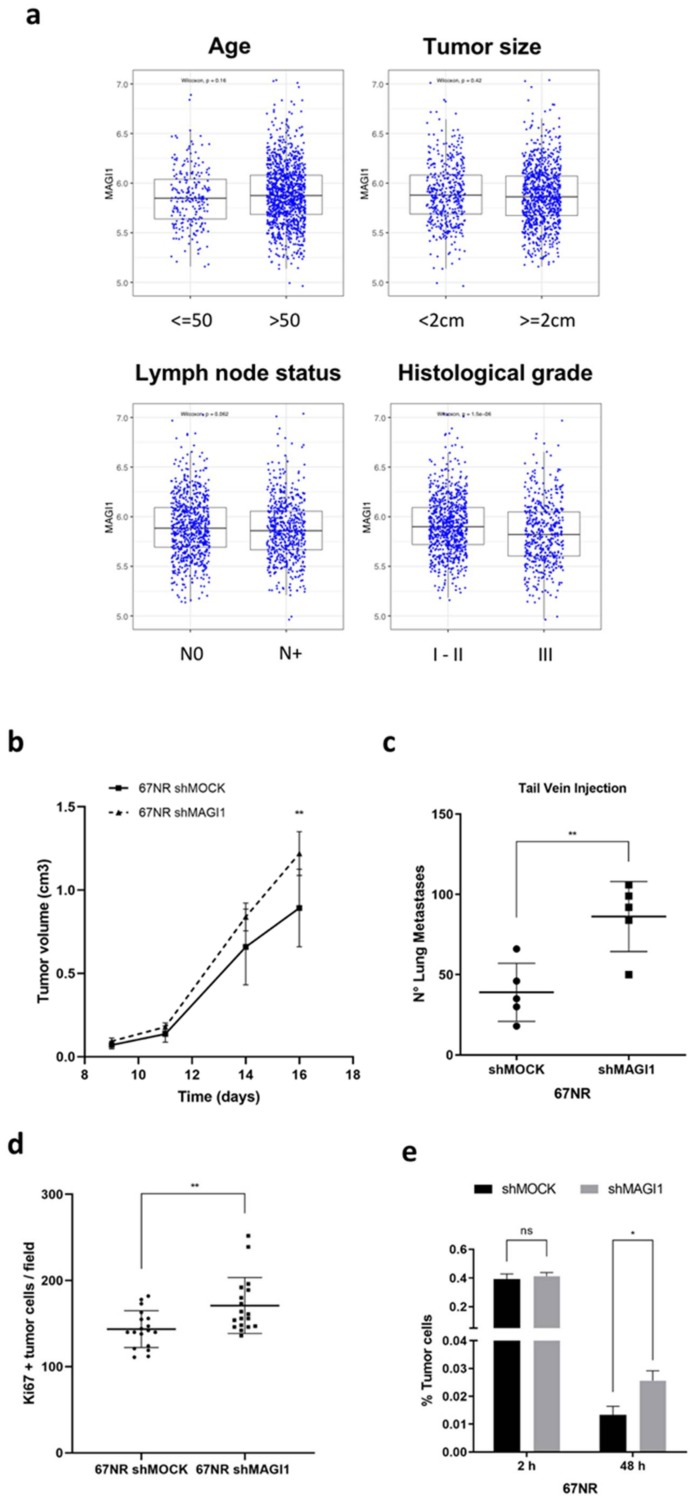
MAGI1 downregulation promotes metastatic progression of ER^+^ cancers. (**a**) MAGI1 expression in ER^+^/HER2^−^ patients from METABRIC microarray analysis by age (≤50 vs. >50), tumor size (<2 cm vs. ≥2 cm), lymph node status (N0 vs. N+) and histological grade (III vs. I–II). Statistical analysis was performed by Kruskal–Wallis test (non-parametric one-way ANOVA). (**b**) MAGI1 silencing enhances growth of primary tumors derived from 67NR cells. Data represent mean values ± S.D (*n* = 7 mice). (**c**) MAGI1 silencing enhance lung metastatic nodules number in BALB/c mice injected via tail vein with 67NR (shMOCK and shMAGI1) cells. Data represent mean values ± S.D. (*n* = 5 mice). (**d**) Quantification of Ki67 staining of lung metastatic nodules of BALB/c mice injected via tail vein with 67NR (shMOCK and shMAGI1) cells. Ki67 positive cells were counted per high-power field (63× objective). The data represents mean ± S.D. (*n* = 18 fields per condition). (**e**) Quantification of tumor cells present within the lungs 2 h (2h) or 48 h (48h) after tail vein injection of 67NR (shMOCK and shMAGI1) cells. (*n* = 3 mice). Statistical analyses were performed by unpaired *t*-test. ns = no statistical difference, * *p* < 0.05, ** *p* < 0.01.

Among the standard clinical and pathological variables, i.e., age (≤50 vs. >50), tumor size (<2 cm vs. ≥2 cm), lymph node status (N0 vs. N+) and histological grade (I–II vs. III), only histological grade shows a statistically significant differential expression of MAGI1 consistent with the above histopathological observations (Figure 5). Patients with grade 3 tumors have lower MAGI1 expression compared to low grade tumors (Figure 6a). This observation is further consistent with the negative correlation between MAGI1 expression and the histological grade signature (GGI grading) in ER^+^/HER2^−^ BC observed at the transcriptomic level in the METABRIC and TCGA datasets (Figure 1b). There was also a trend (*p* = 0.062) toward lower expression of MAGI1 in patients with positive lymph node (LN) status (Figure 6a). These observations suggest that MAGI1 downregulation in ER^+^/HER2^−^ BC may promote tumor progression.

To experimentally assess whether MAGI1 downregulation increased tumor progression, we set up to perform in vivo tumor growth and metastasis assays. For this we used two sister-cell lines derived from a mouse adenocarcinoma model in BALB/c [46] with different MAGI1 expression levels (Figure S2a) and metastatic capacities: 67NR (ER^+^) being non-metastatic and 4T1 (ER^−^), highly metastatic [47]. To test whether MAGI1 expression may suppress metastasis, we downmodulated MAGI1 expression in 67NR cells [48] and up-regulated it in 4T1 cells by shRNA and cDNA overexpression, respectively (Figure S4a,b). Cells were then orthotopically injected in the fat pad of the 4th mammary gland and monitored for tumor growth and lung metastatic burden. Downregulation of MAGI1 in 67NR cells slightly but significantly increased tumor growth (Figure 6b), however we could not detect neither micro- nor macro-metastasis in the lungs. Given the non-metastatic phenotype of these cells it is not surprising that downregulation of MAGI1 alone was not sufficient to allow cancer cells to accomplish the complete metastatic cascade from the primary tumor. Overexpression of MAGI1 in the 4T1 cell line did not affect tumor growth (Figure S4c), nor it reduced spontaneous lung metastasis formation (Figure S4d). In order to experimentally test MAGI1 effect on metastatic potential in 67NR cells, we performed an experimental lung metastasis assays, by injecting 67NR cells through the tail vein. Interestingly, 67NR cells with downregulated MAGI1 generated a higher number of metastatic nodules in the lungs compared to 67NR shMOCK cells (Figure 6c). Lung metastatic nodules originated from 67NR with downregulated MAGI1 had a higher fraction of proliferating cells than controls, as determined by Ki67 IHC staining (Figure S4e and Figure 6d). Consistent with these observations, 67NR cells with downregulated MAGI1 had a growth advantage in vitro compared to control cells (Figure S4f).

Seeding and initial survival in the target organ is a rate-limiting step in metastasis formation [49]. To test whether MAGI1 may impinge on seeding and initial survival of cancer cells disseminated in the lung, we injected fluorescently labeled 67NR cells through the tail vein and sacrificed mice after 2 h (to assess the fraction of cells retained in the lung) and 48 h (to assess the fractions of cells initially surviving in the lung). Two hours after injection there was no difference in the number of cells recovered from the lungs, while at 48 h there were about twice as many viable 67NR cells with silenced MAGI1 than control cells (Figure 6e).

Taken together, these results indicate that low levels of MAGI1 promote lung metastasis of ER^+^ 67NR cells and this effect is associated with enhanced early survival (i.e., 48 hours’ time point) of disseminated cancer cells. MAGI1 silenced 67NR cells also form slightly larger primary tumors and are more proliferative in vitro and in the metastatic nodules.

### 2.6. Low MAGI1 Expression Predicts Poor Prognosis in ER^+^ Breast Cancer

**Figure 7:**
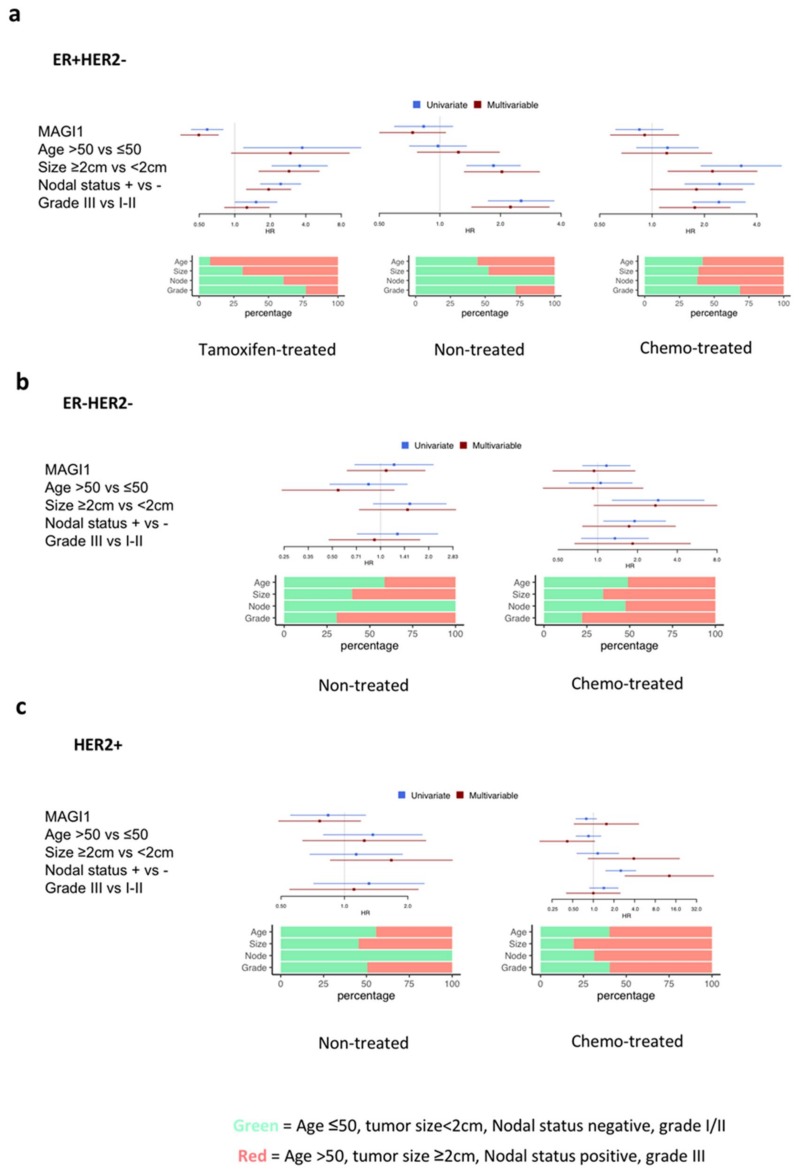
Low MAGI1 expression predicts poor prognosis in ER^+^ breast cancer. (**a**–**c**) Forest plots showing correlation of MAGI1^high^, age, tumor size, nodal status and grade with risk of relapse (hazard ratio, HR) in (**a**) ER^+^/HER2^−^, (**b**) ER^−^HER2^−^, and (**c**) HER2^+^ BC patients. Datasets retrieved from Haibe-Kains et al., 2012 [50]. MAGI1 expression has been considered as a continuous variable. Statistical analyses were performed with Cox proportional hazards univariate and multivariate models.

To validate whether these experimental in vitro and in vivo observations were of clinical relevance, we collected correlative evidence in BC patients. To this end, we investigated the prognostic value of MAGI1 mRNA level in three historical cases series with long-term follow-up and available gene expression data [50]. In the first set, patients did only receive endocrine treatment (tamoxifen), in the second, the patients did not receive any type of systemic treatment and in the third, patients were treated with chemotherapy and endocrine therapy. The detailed clinical-pathological characteristics of the patients in these three datasets are given in Table S3. The distribution of the standard clinical variables differs between these three datasets. Since we demonstrated a differential MAGI1 expression between ER^+^/HER2^−^, HER2^+^, and ER^−^/HER2^−^ tumors (Figure 1a), we investigated the prognostic value of MAGI1 expression in each of these subtypes. We found that high MAGI1 expression is associated with a lower risk of relapse in ER^+^/HER2^−^ BC treated with tamoxifen at the univariate (HR 0.58 [0.43–0.80], q = 0.001), and multivariate level (HR 0.50 [0.34–0.73], q = 0.001). The prognostic value of MAGI1 for disease free survival (DFS) was however absent in the untreated (composed mainly of low-grade patients without axillary lymph-node involvement) and chemotherapy-treated dataset (characterized by high-risk features such as lymph node involvement and high grade in more than half of the patients) (Figure 7a). In contrast, in the HER2^+^ and ER^−^/HER2^−^ subgroups, there was no association between MAGI1 expression levels and DFS in any of the datasets (Figure 7b,c).

To assess how low MAGI1 expression levels in ER^+^ BC patients compare to genomic tests in predicting the risk of recurrence, we used the 21 gene recurrent score (GENE21) [51] as a surrogate for the OncotypeDX recurrence score [52]. When we added GENE21 to the multivariable Cox regression model (which includes MAGI1, age, grade and nodal status), both GENE21 and MAGI1 were significant and anti-correlated in the tamoxifen dataset, meaning that these two variables are independently associated with DFS (i.e., they provide independent information) in the tamoxifen dataset (Figure S5a). Next, we investigated the impact of GENE21 on the prognostic effect of MAGI1 in untreated and chemotherapy-treated ER^+^/HER2^−^ patients. While increased GENE21 score was associated with increased risk of relapse in both untreated and chemotherapy-treated patients, the effect of MAGI1 still remained non-significant in the multivariable models including GENE21 (Figure S5b,c).

Altogether, these results show that MAGI1 provides prognostic information non-redundant relative to genomic information, in particular for tamoxifen treated patients.

### 2.7. MAGI1 Expression Negatively Correlates with Inflammation in ER^+^ Breast Cancer and Is Downregulated by the Prostaglandin E2 (PGE2)/Cyclooxygenase-2 (COX-2)axis

**Figure 8:**
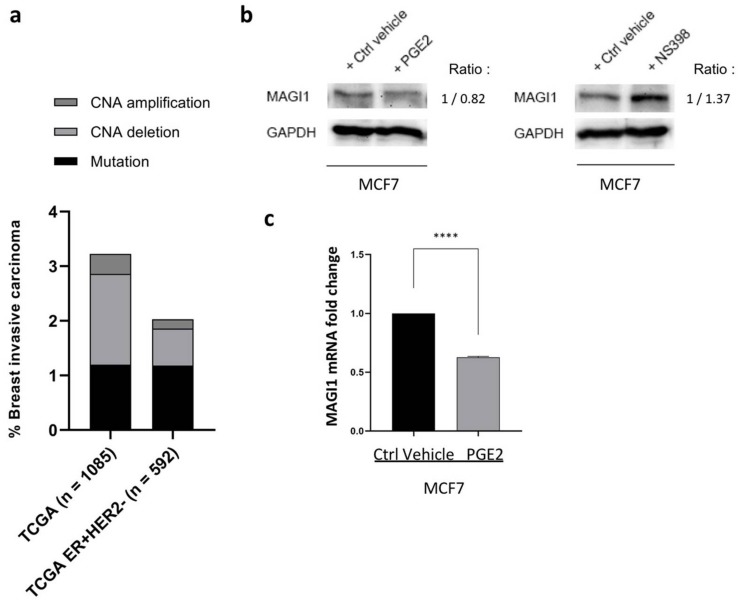
MAGI1 expression negatively correlates with inflammation in ER+ breast cancer and is modulated by prostaglandin E2 (PGE2)/COX-2axis. (**a**) Plot representing percentages of BC patients with MAGI1 mutations and copy number alterations (CNAs) in TCGA datasets. TCGA, *n* = 1085 (Mutations = 1.198%, Deletions = 1.658%, Amplifications = 0.368%). TCGA ER^+^HER2^−^, *n* = 592 (Mutations = 1.182%, Deletions = 0.675%, Amplifications = 0.168%). (**b**) Western blot showing MAGI1 protein levels in MCF7 cells treated with 5 µM of PGE2 and 100 µM NS-398 (COX-2 inhibitor) and corresponding vehicle control (DMSO). GAPDH is used as loading control. Band intensity ratio adjusted to GAPDH is shown next to the blot. (**c**) Real time PCR quantification of MAGI1 mRNA in MCF7 cells treated with either vehicle control (DMSO) or 5 µM PGE2. Data are shown as a percentage of GAPDH and represent mean values ± SD (*n* = 3 independent experiments, each analyzed in triplicate). Statistical analysis was performed by unpaired *t*-test. ns = no statistical difference, **** *p* < 0.001.

Considering its clinical relevance, one important emerging question concerns the mechanisms of downregulation of MAGI1 expression in ER^+^ BC. We interrogated the TCGA dataset to investigate whether MAGI1 expression could be influenced by DNA alterations. The results showed that MAGI1 mutations or copy number alterations (CNA) are rare as they are observed in less than 3% of all breast cancers, and 2% within ER^+^/HER2^−^ BC (Figure 8a), indicating that DNA alterations are not the major cause of MAGI1 downregulation.

We have previously shown that in colorectal cancer, MAGI1 expression is negatively or positively regulated by PGE2 or COX-2 inhibitors (COXIBs), respectively [4]. Taking into consideration the role of chronic inflammation and PGE_2_ in cancer development and progression, including in BC [53,54,55,56], we therefore sought to study whether MAGI1 expression was possibly modulated by PGE2 or COXIB in BC. Indeed, treatment of MCF7 cells with PGE2 and NS-398, a selective COX-2 inhibitor, lead to downregulation and upregulation of MAGI1 protein levels, respectively (Figure 8b). Downregulation by PGE2 was further confirmed at mRNA level by RT-PCR (Figure 8c). Importantly, in the METABRIC and TCGA datasets we observed that in ER^+^/HER2^−^ BC MAGI1 expression negatively correlates with immune-inflammatory cellular signatures and with the expression of the monocyte/macrophage attracting chemokines CCL2 and CCL7 (Figure S6). GO analysis of human data sets revealed that MAGI1 expression is strongly negatively correlated with biological processes involved in immune and inflammatory reactions (GO:0002376, q = 3.53x10^−77^) including leukocyte and lymphocytes activation, proliferation and cytokine signaling (Figure S1b and Table S2).

These results indicate that MAGI1 expression negatively correlates with immune and inflammatory gene signatures and processes. Experimentally, the inflammatory mediator PGE2 downregulates MAGI1 in MCF7 cells, while COX-2 inhibition upregulates it.

## 3. Discussion

In this study, we report three main sets of findings: Firstly, in human BC, MAGI1 expression is higher in the ER^+^/HER2^−^ subset, and within this subtype low MAGI1 levels correlate with a worse prognosis. Secondly, experimental data demonstrate that downregulation of MAGI1 in ER^+^/HER2^−^ cancer cells generates a more aggressive cancer cell phenotype. Thirdly, reduced MAGI1 expression in ER^+^/HER2^−^ cancer correlates with inflammation, based both on human transcriptomic and experimental data.

We observed that high MAGI1 expression is associated to ER^+^/HER2^−^ BC subtype while low MAGI1 expression within this subtype predicts a more aggressive behavior. Among ER^+^/HER2^−^ BC patients, high MAGI1 expression positively correlates with ESR1 and the luminal genes GATA3 and FOXA1. Estradiol treatment of ER^+^ MCF7 cells upregulates MAGI1 expression. MAGI1 silencing impairs ESR1 expression and signaling, promotes cell proliferation and reduces apoptosis. Clinically, loss of ER expression and increased proliferation in luminal ER^+^ BC patients correlates with a decreased survival, and predicts resistance to endocrine therapy and benefits from chemotherapy [48,57,58]. MAGI1 silencing in ER^+^ MCF7 cells increases phosphorylation of AKT (activation) and GSKβ-3 (inactivation) and Axin2 expression demonstrating increased AKT-Wnt signaling. These results suggest that loss of MAGI1 expression in ER^+^ BC may promote proliferation and survival though the PI3K-Wnt pathway independently from ER signaling. These observations raise the question whether low MAGI1 expression in ER^+^ BC may correlate with high AKT signaling and resistance to ER antagonists. This is of potential clinically relevance, as the PIK3CA inhibitor alpelisib in combination with fulvestrant in progressing ER^+^HER2^−^ BC with mutated PI3KCA, provides survival advantages [18].

We found that high levels of MAGI1 correlates with a lower risk of relapse in ER^+^/HER2^−^ patients, especially in those treated with tamoxifen compared to untreated or chemotherapy-treated patients. Conversely, ER^+^ patients treated with chemotherapy have higher grade tumors, more positive LN and are at higher risk of recurrence [59]. Currently available gene expression signatures, such as OncotypeDX [52], identify patients that could be safely spared of chemotherapy or patients at high risk that need chemotherapy, however, they fall short of value in the intermediate score cases [25,26,33,60]. Interestingly, high MAGI1 level and high GENE21 signature [51] were significantly anti-correlated in the tamoxifen dataset, meaning that these two variables provide independent information relative to DFS in these patients. The effect of MAGI1 in the multivariable models including the GENE21 signatures was non-significant in the untreated and chemotherapy treated groups. Altogether, these results indicate that MAGI1 provides complementary information to currently existing prognostic genomic tools, in the tamoxifen treated patients. Low MAGI1 expression potentially identifies a subset of ER^+^ patients treated with tamoxifen with a yet unidentified increased risk of recurrence.

We further observed that low MAGI1 expression is associated with higher histological grade, increased cell proliferation and reduced epithelial differentiation suggesting that MAGI1 expression is downregulated during tumor progression, consistent with previous reports [4,5,6]. Higher histological grade is positively associated with increased risk of metastatic progression [61]. Consistently, in the 67NR ER^+^ murine BC model, MAGI1 downregulation increases lung metastasis formation possibly promoting tumor cell extravasation, initial seeding and/or early survival in the lung preceding metastatic niche formation [62,63,64]. In contrast, MAGI1 expression levels do not impact TNBC patient prognosis, a patient subgroup characterized by high-grade tumors, and experimental upregulation of MAGI1 in TNBC-like 4T1 tumor cells does not reduce their metastasis capacity.

A main arising question concerns the mechanism of MAGI1 downregulation in ER^+^/HER2^−^ BC cells. MAGI1 gene mutations or copy number alterations appear not to be involved. Strikingly, MAGI1 expression negatively correlates with inflammatory/immune signatures in patients, and PGE2 or COXIB treatment of ER^+^ MCF7 cells reduces or enhances MAGI1 expression, respectively. We have previously reported similar observations in colorectal cancer cells [4]. Considering that chronic inflammation is associated with an increased risk of cancer incidence and progression [65], this is a highly clinically relevant observation. COX-2, the rate-limiting enzyme in PGE_2_ synthesis, is overexpressed in several cancers including BC [53,57]. PGE2 stimulates tumor cell proliferation, invasion, and angiogenesis, inhibits tumor cell apoptosis, and promotes immunosuppression [54,66,67]. Preclinical and clinical studies showed that nonsteroidal anti-inflammatory drugs (NSAIDs) have chemopreventive effects on several cancers including BC [68,69,70], and exert anti-tumor activities, and in particular ketorolac when administrated in the peri-operative setting [65,71]. However, their routine use as adjuvant treatment in BC is still inconsistent and not yet recommended [72,73,74,75,76,77,78,79]. Of interest, mounting evidence indicate that inflammation may promote acquired endocrine resistance and more aggressive progression or ER^+^ BC [80,81,82,83]. Whether MAGI1 is part of this mechanism, and whether this protein is possibly regulated by other tumor-promoting mediators of inflammation (e.g., TNF, IL6) is an attractive hypothesis that deserves further investigations.

## 4. Materials and Methods

### 4.1. Cell Lines

The human MCF7 and MDA-MB-231 BC cell lines were obtained from the American Type Culture Collection (ATCC, Manassas, WV, USA) and the murine BC cell lines 4T1, 67NR were obtained from Dr. Fred Miller (Michigan Cancer Foundation, Detroit, MI, USA). Cells were cultured in Dulbecco’s modified Eagle’s medium (DMEM) supplemented with 10% FBS, 1% penicillin/streptomycin, and maintained in a humidified incubator at 37 °C and 5% CO_2_.

### 4.2. Cell Treatments

For PGE_2_ or COX-2 inhibitor treatment, MCF7 cells were treated for 48 h with 5 µM PGE2 (Enzo Life Sciences, Lausen, Switzerland), or for 72 h with 100 µM NS-298 (inhibitor of COX-2) (Enzo Life Sciences). DMSO was used as a control vehicle for both treatments. For 17-β-estradiol treatment, MCF7 cells were serum starved for 48 h in phenol-red-free DMEM/F-12 media (SFM). Cells were treated for 6 h with either 10^−6^ M 17-β-estradiol (Sigma-Aldrich, St. Louis, MO, USA) or a vehicle control (ethanol, 0.1%). For ER inhibition experiments, 10 µM 4-Hydroxi-Tamoxifen (Tocris Bioscience, Cat. No. 3412, Bristol, UK) or 0.5 µM ICI 182,780 (Tocris Bioscience, Cat. No. 1047) were added to cells 1 h before 10^−6^ M 17-β-estradiol and further cultured for another 6 h as above. For the control condition cells were treated with ethanol 0.1%. After treatments, either protein or total RNA was extracted and western blot or quantitative real-time PCR was performed.

### 4.3. Animal Procedures Authorization

All animal procedures were performed in accordance with the Swiss legislations on animal experimentation and approved by the Cantonal Veterinary Service of the Cantons Fribourg (2014_58_FR and 2017_34FR).

### 4.4. Spontaneous Tumor Models

MMTV-PyMT (FVB/N) mice were bred and housed in ventilated cages in the OHB mouse husbandry of the University of Fribourg. Spontaneously-derived PyMT-MMTV tumors [84] were extracted from 4-month old mice and tumor samples were stored and pulverized in liquid nitrogen. RNA was extracted as described in Supplementary Materials and methods.

### 4.5. Orthotopic Tumor Models

4T1 cells (2.5 × 10^5^ cells in 100 µl PBS) either transduced with the control vector (psD44 MOCK) or with a MAGI1 expressing vector (psD44 MAGI1OE), were injected in the mammary fat pad of the fourth inguinal mammary gland of 7-week-old female BALB/c mice (Envigo, Huntingdon, UK). 67NR cells (4 × 10^5^ cells in 50 µl PBS) either transduced with a control vector (shMOCK or shControl) or with MAGI1 short hairpin RNA expressing vector (shMAGI1), were injected as above in 6-week-old female NSG mice (Animal facility University of Lausanne, Lausanne, Switzerland). To monitor tumor growth, tumor size was measured at regular intervals and tumor volume was calculated with the following equation: Tumor volume = (length × width^2^)/2. At the end of the experiment mice were sacrificed and lungs were resected. Lung macro-metastases at the surface were counted with a stereomicroscope, thereafter lungs were fixed in 4% PFA overnight and then prepared for paraffin-embedding.

### 4.6. Experimental Lung Metastasis Model

67NR cells (25 × 10^4^ cells in 100 µL PBS) either transduced with a control vector (shMOCK) or with MAGI1 short hairpin RNA expressing vector (shMAGI1), were injected via the tail vein into 7-week-old BALB/c female mice (Envigo, Huntingdon, UK). 21 days post-injection mice were sacrificed and lungs were resected for metastasis measurements. Lung macro-metastases at the surface were counted with a stereomicroscope and lungs were incubated overnight in 4% PFA and then paraffin-embedded for sectioning and HE and immunohistochemistry staining. For monitoring the in vivo fate of injected cancer cells, 67NR cells (shMOCK and shMAGI1) were stained with 5 µM green fluorescent dye Cell-Tracer CFSE (Thermo Fisher Scientific, Waltham, MA, USA) following the manufacturer’s instructions. 10^5^ cells were injected via tail vein into 7-week-old BALB/c female mice (Envigo, Huntingdon, UK). Mice were divided into two groups: the first group was sacrificed after 2 h (to quantify tumor cells sequestered in the lungs) and the second group was sacrificed 48 h post-injection (to quantify tumor cell extravasation and early survival into the lungs). When mice were sacrificed, lungs were resected for flow cytometry analysis. Lung samples were disaggregated with a digestion solution composed by 0.4 U/mL Liberase TH Research grade (Roche, Basel, Switzerland) and 25 µg/mL DNase I (DN25) (Sigma-Aldrich, St. Louis, MO, USA), for 1 h at 37 °C. Disaggregated lungs were washed with PBS-EDTA (2 mM) and PBS, and filtered with 70-µm mesh (Biologix, Shandong, China). Finally, red blood cells were lysed with ACK lysis buffer (Thermo Fisher Scientific, Waltham, MA, USA). Samples were filtered again with a 70-µm mesh and analyzed by flow cytometry with MACS Quant Analyzer 10 (Miltenyi Biotec, Bergisch Gladbach, Germany). Data analysis was performed with FlowJo.

### 4.7. Cytoimmunofluorescence Staining

Cells were plated in sub-confluent conditions on glass cover-slips placed in 12 well plates and cultured for 24–48 h in complete media or phenol-red serum-free DMEM/F-12 (SFM). Thereafter, cells were fixed with 4% PFA for 15 min at 4 °C and washed with PBS. To block unspecific binding of the antibodies, cells were incubated with 5% Donkey serum (Sigma-Aldrich) for 1 h at room temperature. Then, samples were incubated with primary antibodies for 1 h at room temperature. After four washings with 1x PBS, samples were incubated with secondary antibodies conjugated to Alexa fluor-488,546,568 or 647 as necessary for 1 h at room temperature in a humid-chamber protected from light. Cell nuclei were counterstained and mounted in ProLongTM Gold antifade reagent with DAPI (Thermo Fisher Scientific). Images were acquired with a Leica TCS SP5 inverted confocal laser scanning microscope using a 40× or 63× objective and with a pinhole of 1 AU to minimize z-section.

### 4.8. Tissue Staining

*Histopathological and immunohistochemical analyses of mouse tissues.* Standard hematoxylin–eosin (HE) and Ki67 staining procedures were performed on paraffin-embedded tissues. Tissue sections were digitalized by the slide scanner Hamamatsu Nanozoomer (Hamamatsu, Japan). Proliferation in Ki67 stained slides were assessed by counting Ki67+ breast cancer cells per field in scanned slides (63× zoom in Nanozoomer).

*Histopathological and immunohistochemical analyses of human tumors.* Stained tumors were part of a Tissue Macro Array (TMA) kindly provided by the Tissuebank Bern (TBB). TMAs were cut at 3 μm sections and mounted onto glass slides, dried and baked at 60 °C for 30 min prior to use. HE staining was performed following standard procedures. Immunohistochemistry was performed by automated staining using Bond RX (Leica Biosystems, Wetzlar, Germany) immunostainer. All slides were dewaxed in Bond dewax solution (Leica Biosystems). Heat-induced epitope retrieval was performed at pH 9 in Tris buffer based (Leica Biosystems) for 30 min at 95°. MAGI1 rabbit polyclonal antibody (Sigma Aldrich, Munich, Germany) was incubated for 30 min at 1:50 dilution. Then samples were incubated with HRP (Horseradish Peroxidase)-polymer for 15 min and subsequent visualized using 3,3-Diaminobenzidine (DAB) as brown chromogen (Leica Biosystems) for 10 min. Finally, the samples were counterstained with Haematoxylin, dehydrated and mounted with PERTEX ® (Histolab, Gothenburg, Sweden). Slides were scanned and photographed using Pannoramic 250 scanner and Case Viewer (3DHistech, Budapest, Hungary). Tissues were used upon approval and in accordance with ethical institutional rules (ethics approval number: KEK 200/2014).

### 4.9. Bioinformatic Analyses—Gene Expression and Survival Analyses

The METABRIC [34] and TCGA dataset [35] including clinical data and normalized gene expression, was retrieved through cBioportal on the 08/04/19 [37]. Subtypes were assessed according to the ER immunohistochemistry and HER2 immunohistochemistry or FISH status. Tamoxifen-treated, systemically untreated node negative patients, and chemotherapy treated patient were retrieved from the dataset in [50]. Gene expression signatures were retrieved from the literature (ESR1_signature, AURKA, PLAU, STAT1 [85]; GGI [86]; SDPP [87]; Immune_Perez [88]; IRM [89]; immune cell signatures [90]; GENE21 [91] and computed as described in [92]. Wilcoxon and Kruskal–Wallis tests were performed to compare continuous to categorical variables of 2 or more categories, respectively. Correlations were assessed using Spearman coefficients. In the heatmap only significant correlations are colored: Red, anti-correlated; blue correlated. Survival analysis were performed with Cox proportional hazards univariate models and multivariable models (corrected for age (>50 vs. ≤50), tumor size (≥2 vs. <2), nodal status (pos vs. neg), grade (III vs. I–II)), all additionally corrected for dataset of origin. *p*-values were two-sided and statistical significance considered for *p* < 0.05. All analyses were performed using R 3.5.2.

### 4.10. Bioinformatic Analyses—Gene Onthology Analyses

Genes were considered associated with MAGI1 expression in TCGA when the spearman coefficients were q-value < = 0.01 according to FDR. Positively and negatively associated genes were analyzed independently. Genes were first analyzed with PANTHER [93] for overrepresentation test in *Homo sapiens* within the complete GO biological process. Significant GO terms (FDR < 0.05) were summarized using REVIGO [94] with default parameters.

### 4.11. Statistical Analysis (Except Bioinformatics Analysis)

Normal distribution of samples was assessed by Shapiro-Wilk test. Data from in vitro and in vivo experiments were analyzed by Student’s *t* test or Mann–Whitney test when applicable. Primary tumor growth was analyzed by a two-way ANOVA. Results were considered significant with at least *p* < 0.05 (*), *p* < 0.01 (**), *p* < 0.005 (***), and *p* < 0.001 (****). Results are expressed as mean ± SD unless otherwise indicated.

## 5. Conclusions

In conclusion, we have identified previously unrecognized features and functions of MAGI1 in ER^+^/HER2^−^ BC: MAGI1 is highly expressed and has tumor suppressor activity in ER^+^/HER2^−^ BC subset. MAGI1 loss within this subset correlates with a more aggressive phenotype and identifies patients at higher risk of recurrence. MAGI1 loss positively correlates with inflammation in patients and COX-2 inhibition upregulates its expression. The present observations reinforce the status of MAGI1 as a potential tumor suppressor downregulated during inflammation and cancer progression and upregulated by COXIBs. Whether and how these observations may translate into improved therapy will require additional studies.

## Supplementary Materials

The following are available online at https://www.mdpi.com/2072-6694/12/1/223/s1, Figure S1: Gene ontology (GO) analysis for biological processes correlated with MAGI1 expression. Figure S2: Effect of MAGI1 modulation in 67NR and 4T1 cells on cell proliferation and sensitivity to chemotherapy in MCF7 and 67NR cells with modulated MAGI1 expression. Figure S3: MAGI1 overexpression in the MDA-MB-231 cell line does not induce ERα expression nor its affects proliferation. Figure S4: Effect of MAGI1 silencing and overexpression on 67NR and 4T1. Figure S5: Prognostic value of MAGI1 ER^+^/HER2 breast cancer patients. Figure S6: Heat plot correlation matrices in ER^+^/HER2^−^ patients from METABRIC and TCGA data. Figure S7: Original blots. Table S1: List of GO terms positively correlated with MAGI1 from a gene ontology analysis for biological processes. Table S2: List of GO terms negatively correlated with MAGI1 from a gene ontology analysis for biological processes. Table S3: Detailed clinical-pathological characteristics of the patients in these three datasets. Supplementary Material and Methods for antibodies, lentiviral constructs, real time PCR, Western blot, apoptosis, cell growth, viability and cell cycle assays, and gene ontology analyses.
