# Supplementary material for: MAGI1, a New Potential Tumor Suppressor Gene in Estrogen Receptor Positive Breast Cancer
Source: Cancers (Basel). 2020 Jan 16;12(1):223. doi: 10.3390/cancers12010223 (PMC7016640; doi:10.3390/cancers12010223)
Supplement: Supplementary file 1 [file cancers-12-00223-s001.zip › cancers-574298-Supplementary Methods and Figures.pdf]

Supplementary Materials: MAGI1, a new potential tumor suppressor gene in estrogen receptor positive breast cancer

Begoña Alday-Parejo, François Richard, Janine Wörthmüller, Tilman Rau, José A. Galván, Christine Desmedt, Albert Santamaria-Martinez and Curzio Rüegg

Supplementary Materials and Methods

Antibodies

For this study the following antibodies were used: Anti-MAGI1 rabbit polyclonal antibodies M5691 and HPA031853 (Sigma-Aldrich, St. Louis, MI, USA), Anti-ERα (D-12) mouse monoclonal sc-8005 (Santa Cruz Biotechnology, Dallas, TX, USA), Anti-GAPDH rabbit G9545 (Sigma-Aldrich), Alexa Fluor 568 Phalloidin (Thermo Fisher Scientific, Waltham, MA, USA), Anti-E-cadherin (24E10) rabbit 3195 (Cell signaling, Danvers, MA, USA), Goat anti-rabbit Alexa Fluor 546 A-11071 (Green in the image, for E-cadherin) (Thermo Fisher Scientific), Anti-Ki67 rabbit ab15580 (Abcam, Cambridge, United Kingdom) and HRP-conjugate goat-anti-rabbit (for anti-Ki67) (DAKO, Glostrup, Denmark).

Lentiviral constructs

Cells were stably transfected by lentivirus containing constructs with either short-hairpin RNA or cDNA for downregulation and overexpression of MAGI1, respectively. Non-silencing NSControl and silencing sh4MAGI1 constructs used to transduce MCF7 cells were purchased from Dharmacon (GE Healthcare, Chicago, IL, USA). All constructs are based on the pGIPZ-CMV lentiviral system and contain mammalian puromycin resistance and a GFP-tag. Sh4MAGI1 (ID: V3LHS_346389) targets all human MAGI1 isoforms. ShMOCK and shMAGI1 constructs used to transduce 67NR cells to target the murine isoform of MAGI1 were designed and produced in the laboratory by molecular cloning. Both constructs contain a mammalian blasticidin S resistance gene. The PsD44MOCK control construct and the psD44MAGI1 expressing construct were produced in the laboratory by molecular cloning. MAGI1 cDNA was obtained from Prof. Yukata Hata (Tokyo Medical and Dental University, Japan). Both constructs use a pRRLSIN.cPPT.PGK (=psD44) lentiviral system and contain mammalian puromycin resistance. PsD44MAGI1 contains cDNA of MAGI1b isoform. For the CRISPR/Cas9 4T1 (CRISPR Control and CRISPR MAGI1), the MAGI sgRNAs were cloned into the lentiCRISPRv2 lentiviral vector as described in [2]. The sgRNA and Cas9 nuclease containing lentiviruses were used to infect 4T1 cells at low MOI and select them with 1 μg/mL puromycin.

Real-time PCR

RNA was extracted using the RNeasy mini Kit (Qiagen, Hilden, Germany). 1 μg of RNA was retro-transcribed to Complementary DNA (cDNA) using M-MLV-RT Reverse transcriptase RNase (H-) (Promega, Madison, WI, USA). Quantitative PCR (qPCR) analysis was performed using SensiFAST SYBR Hi-ROX kit (Bioline, London, United Kingdom) in a StepOnePlus thermocycler (Applied Biosystems, Foster City, CA, USA) and gene expression levels were measured by the $2^{-\Delta\Delta Ct}$ method. The list of primers used:

Gene	Accession N	Forward primer	Reverse primer
Magi1	NM_001286785.1	CAGAAGGGGTCTCCAGATCA	ATCCACACCAGGCACTTCTC
MAGI1	NM_015520	TTCAAGGCCGTCAGACAA	ATGGGGGTAAAGGTTATCCC
Gapdh	NM_001289726	CGTCCCGTAGACAAAATGGT	TCAATGAAGGGGTCGTTGAT
GAPDH	NM_002046	GGACCTGACCTGCCGTCTAG	CCACCACCCTGTTGCTGTAG
Arbp1	NM_007475.5	GATTCGGGATATGCTGTTGG	GTTCTGAGCTGGCACAGTGA
Gata3	NM_008091.3	GCTACGGTGCAGAGGTATCC	AGAGATCCGTGCAGCAGAG
Esr1	NM_001302532.1	TCCGGCACATGAGTAACAAA	CCAGGAGCAGGTCATAGAGG
ESR1	NM_001122742.1	ATGATCAACTGGGCGAAGAG	AGGATCTCTAGCCAGGCACA
PGR	NM_000926	TCCTTACCTGTGGGAGCTGT	CGATGCAGTCATTTCTTCCA
BRCA1	NM_007294	TCCTTACCTGTGGGAGCTGT	CGATGCAGTCATTTCTTCCA
SNAI2	NM_005985.3	TGACCTGTCTGCAAATGCTC	TCGGACCCACACATTACCTT
SNAI1	NM_000459.4	CATCTGAGTGGGTCTGGAGG	CTTCTCTAGGCCCTGGCTG
TWIST1	NM_003380.3	GCTCACGAGCGGCTCAGCTA	AATGACATCTAGGTCTCCGGCCCT
KRT18	NM_000224.3	AGATTGACAATGCCCGTCTT	TGGATGTCGTTCTCCACAGA

Western blot

Proteins were extracted in cooled RIPA buffer containing protease inhibitor mix (Sigma-Aldrich) and the following phosphatase inhibitors: phenylmethylsulfonylfluoride (PMSF), sodium orthovanadate (Na_3VO_4) and Glycerophosphate disodium salt hydrate (BGP). Protein lysates were then sonicated and centrifuged at 13000 rpm for 10 minutes at 4°C and supernatants were collected. Protein concentration was calculated by using a BSA standard curved and Bradford assay (Bio-Rad). Proteins were resolved in reducing conditions by SDS-PAGE (sodium dodecyl sulfate polyacrylamide gel electrophoresis) in 9% polyacrylamide gels. Subsequently, proteins were transferred to PVDF Immobilon-FL membranes (Merck Millipore) typically for 60–90 minutes at 100V and 250 mA. Membranes were then blocked with 5% BSA for 1 hour at room temperature and incubated at 4 °C overnight with the corresponding primary antibodies. Next, membranes were washed and then incubated for 1 hour at room temperature with HRP-coupled secondary antibodies (1/2000; DAKO). Protein detection was performed with short incubation with the ECL reagent Luminata Classico Western HRP substrate (Merck Millipore). The band intensity of proteins was quantified by using ImageJ software (National Institutes of Health, Bethesda, MD, USA), and the relative expression of protein was normalized to the expression of GAPDH Loading control).

Apoptosis assay

MCF7 cells were plated in sub-confluent conditions in 6-well plates and cultured in complete medium. After 48 hours, cells were collected and stained with Annexin-V-APC and DAPI (Life Technologies, Carlsbad, CA, USA) following manufacturer’s instructions. Samples were acquired by flow cytometry with MACS Quant Analyzer 10 (Miltenyi Biotec, Bergisch Gladbach, Germany) and analyzed with FlowJo.

Cell cycle analysis

Click-iT Edu Alexa Fluor 647 Flow Cytometry Assay Kit (Thermo Fisher Scientific) was used to assess the cell proliferation status by flow cytometry. MCF7 cells were plated in sub-confluent conditions in 10 cm-plates in complete medium. After 5 hours, cells were incubated with 10 µM EdU (Thermo Fisher Scientific) in complete medium for 90 minutes, and EdU staining was performed according to manufacturer’s instructions. DNA content was stained with DAPI (1 µg/mL) (Thermo Fisher Scientific) for 30 minutes. Samples were acquired by flow cytometry with MACS Quant Analyzer 10 (Miltenyi Biotec) and analyzed with FlowJo.

Cell growth assay

MCF7 cells (2 × 10^4 cells/well; NSControl and sh4MAGI1), 67NR (10^3 cells/well; shControl and shMAGI1) and 4T1 (10^3 cells/well; CRISPR Control vs CRISPR MAGI1) were seeded in 96-well plates in complete media. After 4 hours, once the cells were adhered to the plate, cells were placed in the IncuCyte FLR live cell analysis system (Essen BioScience, Welwyn Garden City, UK). Cell confluence was measured every hour for a total of 48 hours in the case of MCF7 cells, 210 hours for 67NR and 120 hours for 4T1 cells to estimate cell growths.

MTT cell viability assay

MCF7 cells (2 × 10^4 cells/well) and MDA-MB-231 cells (1 × 10^4 cells/well) were seeded in 96-well plates and cultured in complete medium for 24, 48 and 72 hours. Cell viability assay was measured by adding 10% MTT (3-(4,5-dimethythiazol-2-yl)-2,5- diphenyl tetrazolium bromide, 5 mg/mL, Sigma) in complete medium for 4 hours. Then cells were washed once with PBS and absorbance was measured at 570 nm with a spectrophotometry (TECAN infinite M200PRO, Männedorf, Switzerland).

Cell treatment with chemotherapy drugs

MCF7 (NSControl and sh4MAGI1) and 67NR (shControl and shMAGI1) cells were seeded at a concentration of 2000 cells/well in a 96-well plate and grown for 24 hours. The different drugs were added to the cells at a concentration ranging from 0.077 ng/mL to 1200 ng/mL in the case of methotrexate and 0,164 ng/ml to 2560 ng/mL in the case of doxorubicin. Cells were incubated for 72 hours with the compounds and then fixed with 4% PFA for 30 minutes and stained with 0.5% crystal violet for 15 minutes. The plates were washed with distilled water and air-dried overnight at room temperature. Next day the crystal violet was eluted using an eluting buffer solution (70% ethanol and 1% acetic acid) and signals were quantified by measuring the absorbance at 590 nm with the TECAN infinite M200PRO instrument. The average IC50 values from three separate experiments were determined by non-linear regression analysis using the software GraphPad Prism version 8.1.1.

ERE-half sites analysis in promoter sequence

MAG1 promoter sequence was obtained with AceView (NCBI) and ERE half-site motifs in MAGI1 promoter sequence were identified manually as 5′ GGTCA 3′ or 5′ TGACC 3′.

Supplementary Figures

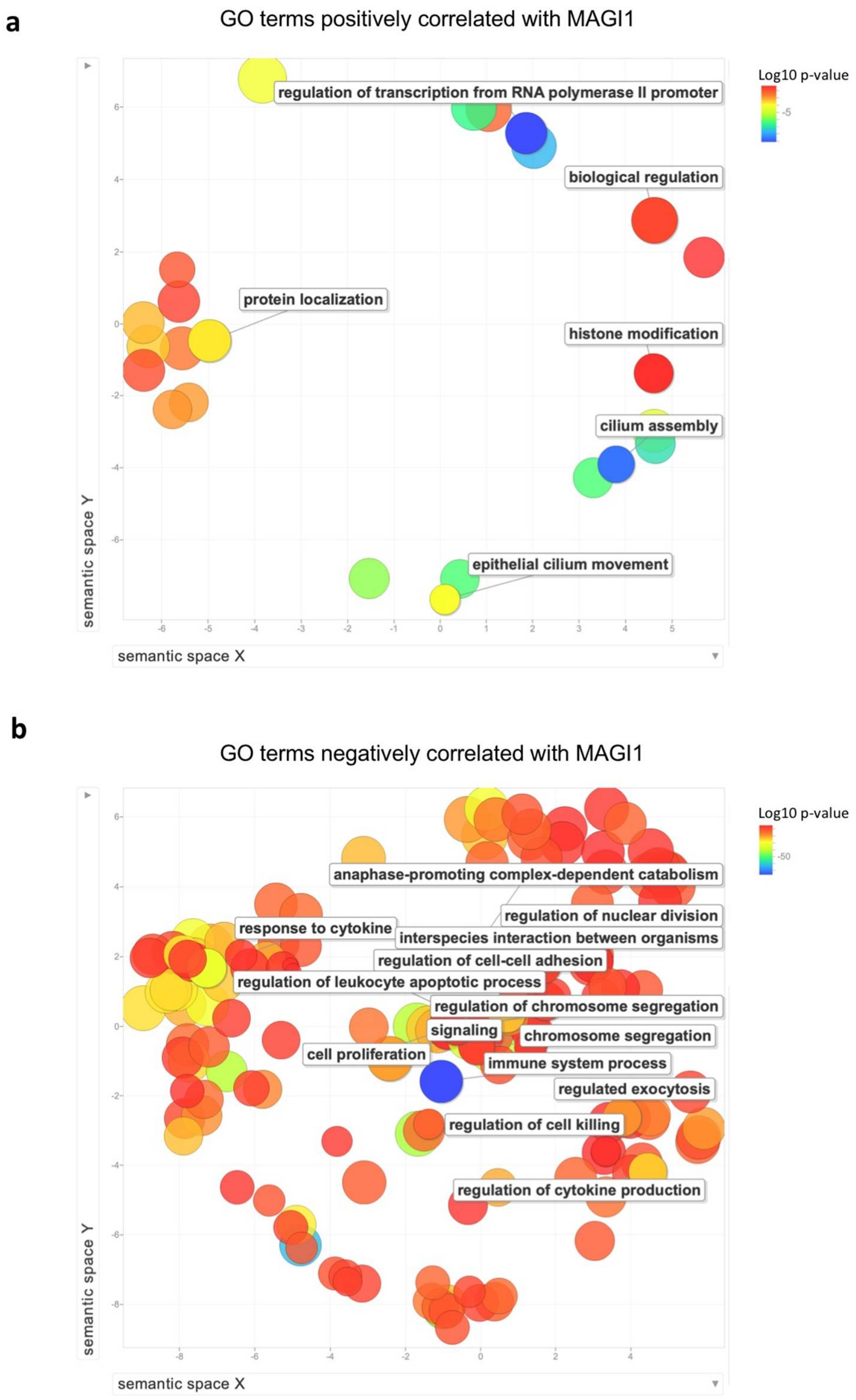

Figure S1. Gene ontology (GO) analysis for biological processes correlated with *MAGI1* expression. (**a**) GO terms positively correlated with MAGI1. (**b**) GO terms negatively correlated with *MAGI1*. Only GO terms that reach statistical significance are included. Statistical significance as FDR < 0.05. Blue depicts strongest significance and red lowest significance.

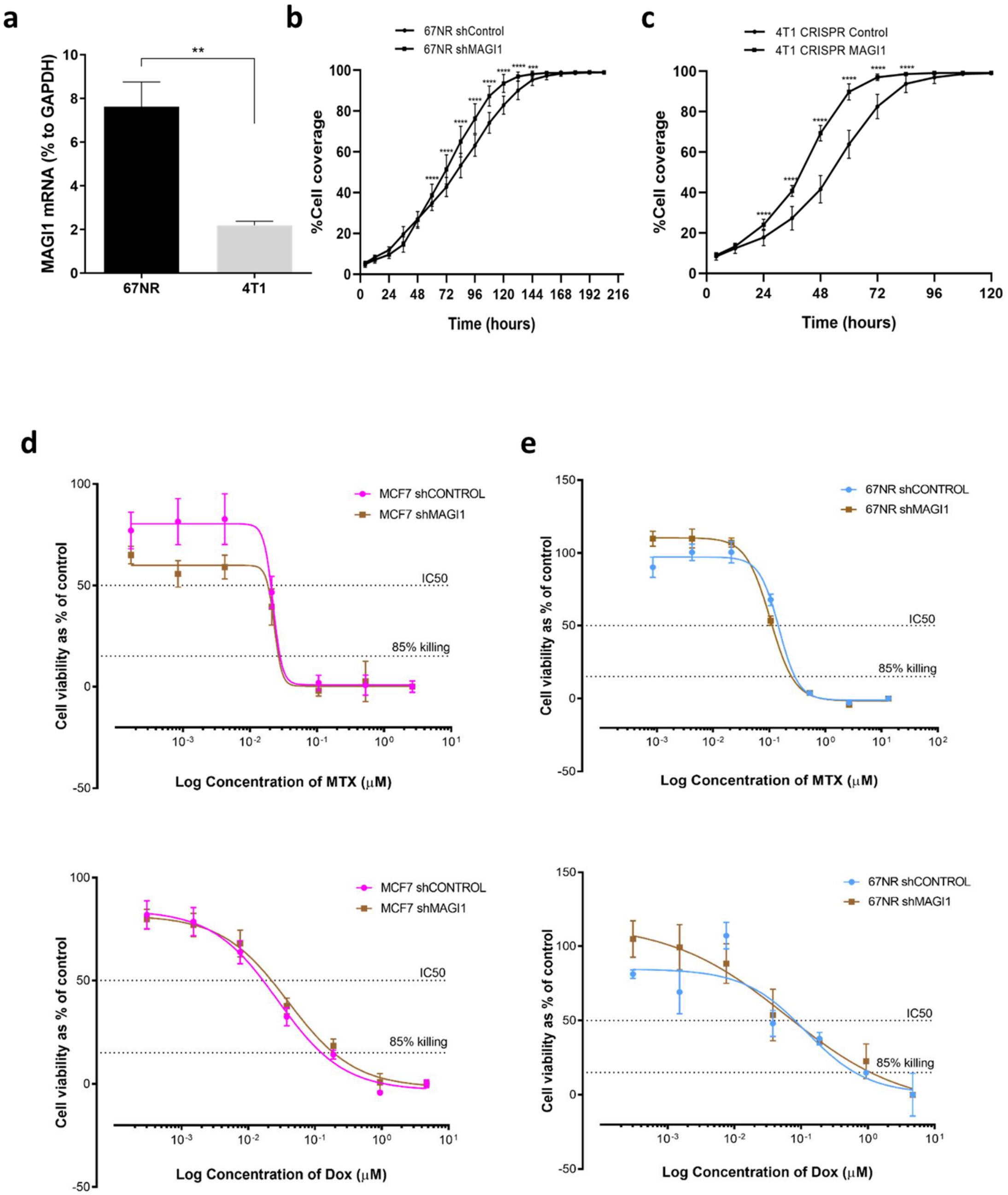

Figure S2. Effect of MAGI1 modulation in 67NR and 4T1 cells on cell proliferation and sensitivity to chemotherapy in MCF7 and 67NR cells with modulated MAGI1 expression. (**a**) Real time PCR quantification of MAGI1 in 67NR and 4T1 cells. Data are shown as percentage of the value of the housekeeping gene GAPDH, and it represents mean values ± S.D. (n = 3 independent experiments, each analyzed in triplicate). (**b–c**) Cell growth curves of (**b**) 67NR shControl and shMAGI1 and (**c**) 4T1 CRISPR Control and CRISPR MAGI1 cells measured as percentage of cell culture well coverage by the IncuCyte system at the indicated time points after plating (n = 24 replicates for 67NR and 18 replicates for 4T1). (**d–e**) Determination of the half-maximal inhibitory concentration (IC50) in (**d**) MCF7 and (**e**) 67NR (shControl and shMAGI1) cells exposed to the chemotherapy drugs methotrexate and doxorubicin at the indicated concentrations. The results are shown as fitted curves averaged from three independent experiments and the IC50 values for each drug are shown in the individual figures (n = 6 replicates per experiment).

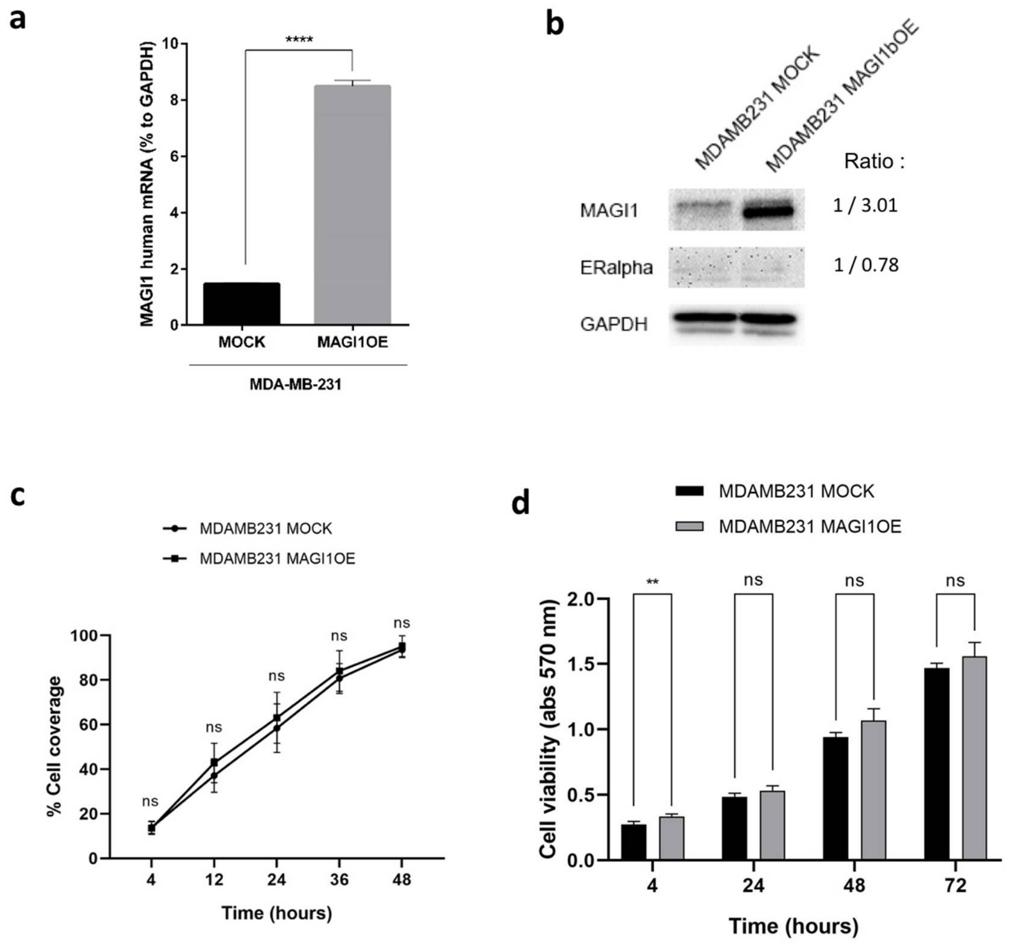

Figure S3. MAGI1 overexpression in the MDA-MB-231 cell line does not induce ERα expression nor its affects proliferation. (**a**) Real time PCR quantification for the validation of MAGI1 overexpression in MDA-MB-231 cells. Data are shown as percentage of the value of the housekeeping gene GAPDH, and represent mean values ± S.D. (n = 3 independent experiments, each analyzed in triplicate). (**b**) Western blot showing MAGI1 and ERα protein levels in MDA-MB-231MOCK and MDA-MB-231MAGI1bOE cells. GAPDH is used as loading control. Band intensity ratio adjusted to GAPDH is shown next to the blot. (**c**) Cell growth of MDA-MB-231MOCK and MDA-MB-231MAGI1bOE cells measured as percentage of cell culture well coverage by the IncuCyte system at the indicated time points (hours) after plating. Data represent mean values ± S.D. (n = 12 replicates). (**d**) Cell viability of MDA-MB-231MOCK and MDA-MB-231MAGI1bOE cells measured by MTT assay at the indicated time points (hours) after plating. Data represent mean values ± S.D. (n = 5 replicates). Statistical analyses were performed by unpaired t-test. ns = no statistical difference, * $p < 0.05$, *** $p < 0.005$, **** $p < 0.001$.

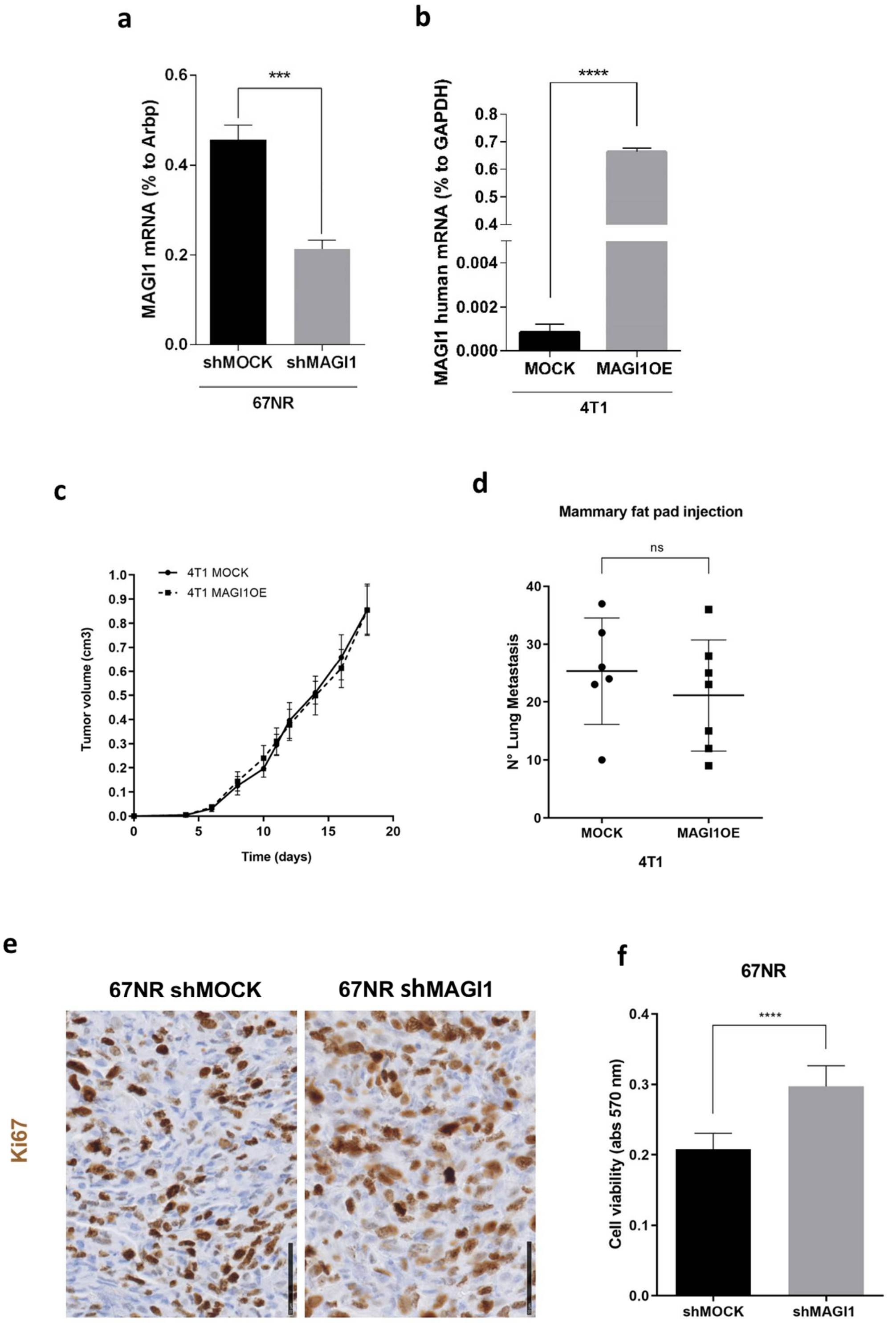

Figure S4. Effect of MAGI1 silencing and overexpression in 67NR and 4T1. (**a**–**b**)**.** Real time PCR quantification of *MAGI1* mRNA for the validation of (**a**) MAGI1 silencing in 67NR cells and (**b**) MAGI1 overexpression in 4T1 cells. Data are shown as a percentage of GAPDH or Arbp1 and represent mean values ± S.D. (*n* = 3 independent experiments, each analyzed in triplicate). (**c**–**d**) MAGI1 overexpression in 4T1 cells (4T1 MAGI1OE) does not affect (**c**) primary tumor growth (**d**) nor spontaneous lung metastasis formation in BALB/c mice compared to control cells (4T1 MOCK). Celle were injected orthotopically in the 4th mammary fat pad. The data represents mean values ± S.D. (*n* =

7 mice). (**e**) Representative high-power images of Ki67 IHC staining of lung metastatic nodules in BALB/c mice injected via tail vein with 67NR (shMOCK and shMAGI1) cells (63× objective). Scale bars correspond to 50 µm. (**f**) Cell viability of 67NR shMOCK and 67NR shMAGI1 cells measured by MTT assay 5 hours after plating. Data represent mean values ± S.D. (n = 3 independent experiments). Statistical analyses were performed by unpaired t-test. ns = no statistical difference, * $p < 0.05$, *** $p < 0.005$, **** $p < 0.001$.

15.6c

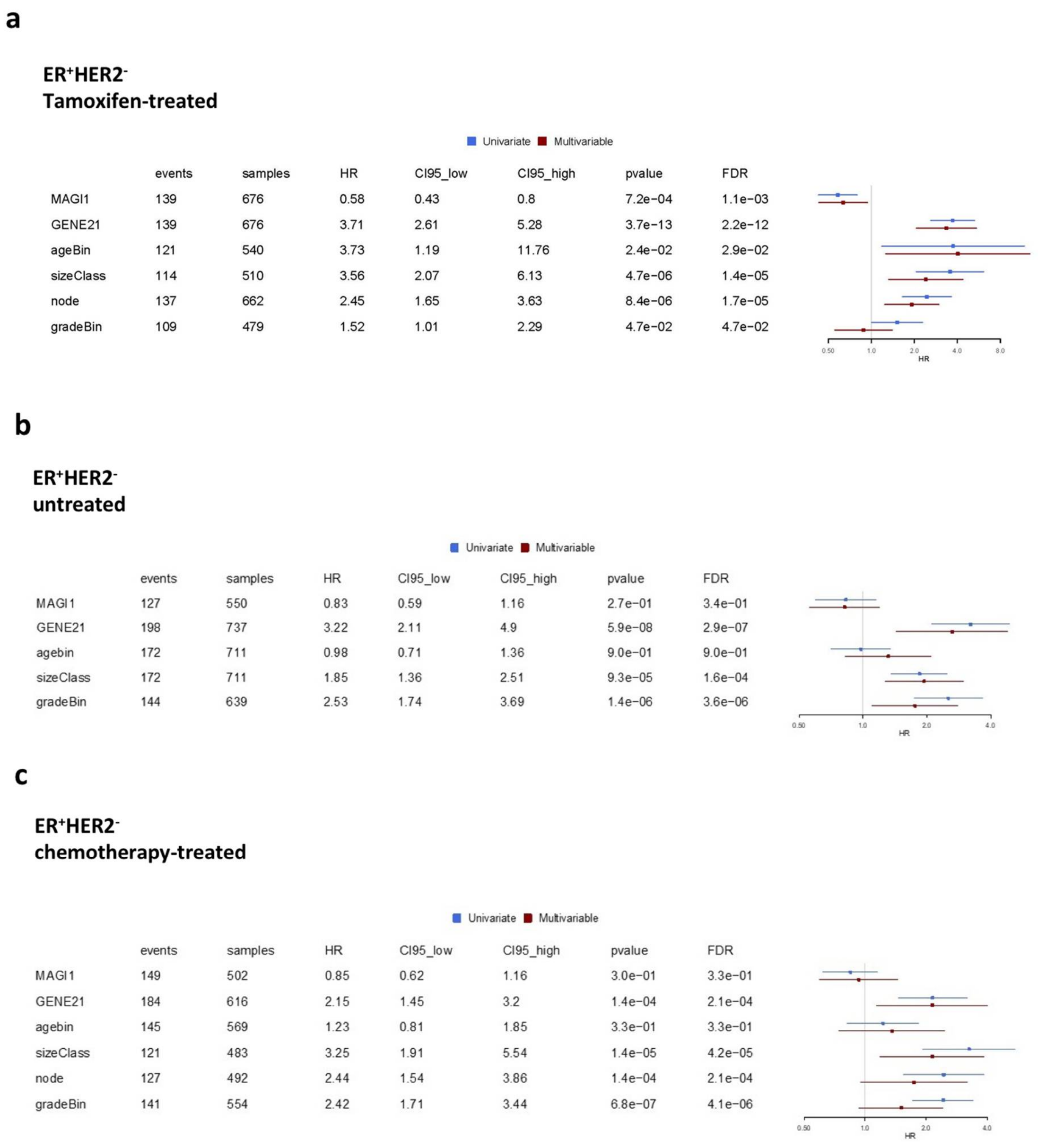

	events	samples	HR	CI95_low	CI95_high	pvalue	FDR
MAGI1	139	676	0.58	0.43	0.8	7.2e−04	1.1e−03
GENE21	139	676	3.71	2.61	5.28	3.7e−13	2.2e−12
ageBin	121	540	3.73	1.19	11.76	2.4e−02	2.9e−02
sizeClass	114	510	3.56	2.07	6.13	4.7e−06	1.4e−05
node	137	662	2.45	1.65	3.63	8.4e−06	1.7e−05
gradeBin	109	479	1.52	1.01	2.29	4.7e−02	4.7e−02

	events	samples	HR	CI95_low	CI95_high	pvalue	FDR
MAGI1	127	550	0.83	0.59	1.16	2.7e−01	3.4e−01
GENE21	198	737	3.22	2.11	4.9	5.9e−08	2.9e−07
agebin	172	711	0.98	0.71	1.36	9.0e−01	9.0e−01
sizeClass	172	711	1.85	1.36	2.51	9.3e−05	1.6e−04
gradeBin	144	639	2.53	1.74	3.69	1.4e−06	3.6e−06

	events	samples	HR	CI95_low	CI95_high	pvalue	FDR
MAGI1	149	502	0.85	0.62	1.16	3.0e−01	3.3e−01
GENE21	184	616	2.15	1.45	3.2	1.4e−04	2.1e−04
agebin	145	569	1.23	0.81	1.85	3.3e−01	3.3e−01
sizeClass	121	483	3.25	1.91	5.54	1.4e−05	4.2e−05
node	127	492	2.44	1.54	3.86	1.4e−04	2.1e−04
gradeBin	141	554	2.42	1.71	3.44	6.8e−07	4.1e−06

Figure S5. Prognostic value of MAGI1 ER+/HER2 breast cancer patients. Forest plots showing correlation of MAGI1^{high}, GENE21, age, tumor size, nodal status and grade with risk of relapse (hazard ratio, HR) in ER+/HER2− BC patients either (**a**) treated with tamoxifen, (**b**) non-treated and (**c**) chemotherapy-treated. Datasets retrieved from [1]. MAGI1 high expression has an independent prognostic value to the prognostic value of GENE21 (a pseudo recurrence score for OncotypeDX) considering DFS in the tamoxifen dataset. Statistical analyses were performed with Cox proportional hazards univariate and multivariate models.

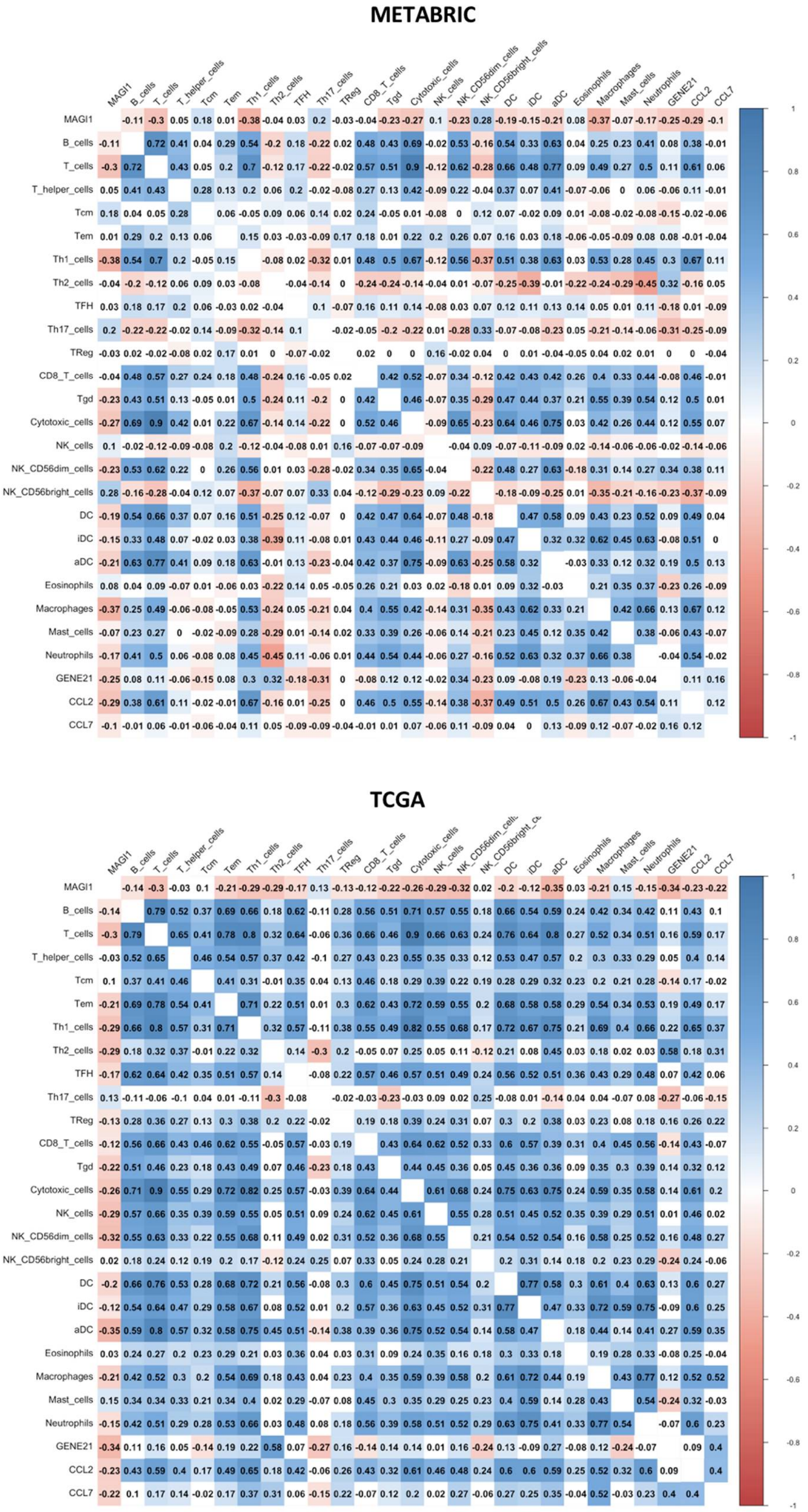

Figure 6. Heat plot correlation matrices in ER+/HER2- patients from METABRIC and TCGA data. Significant correlations are in blue, anti-correlations are in red.

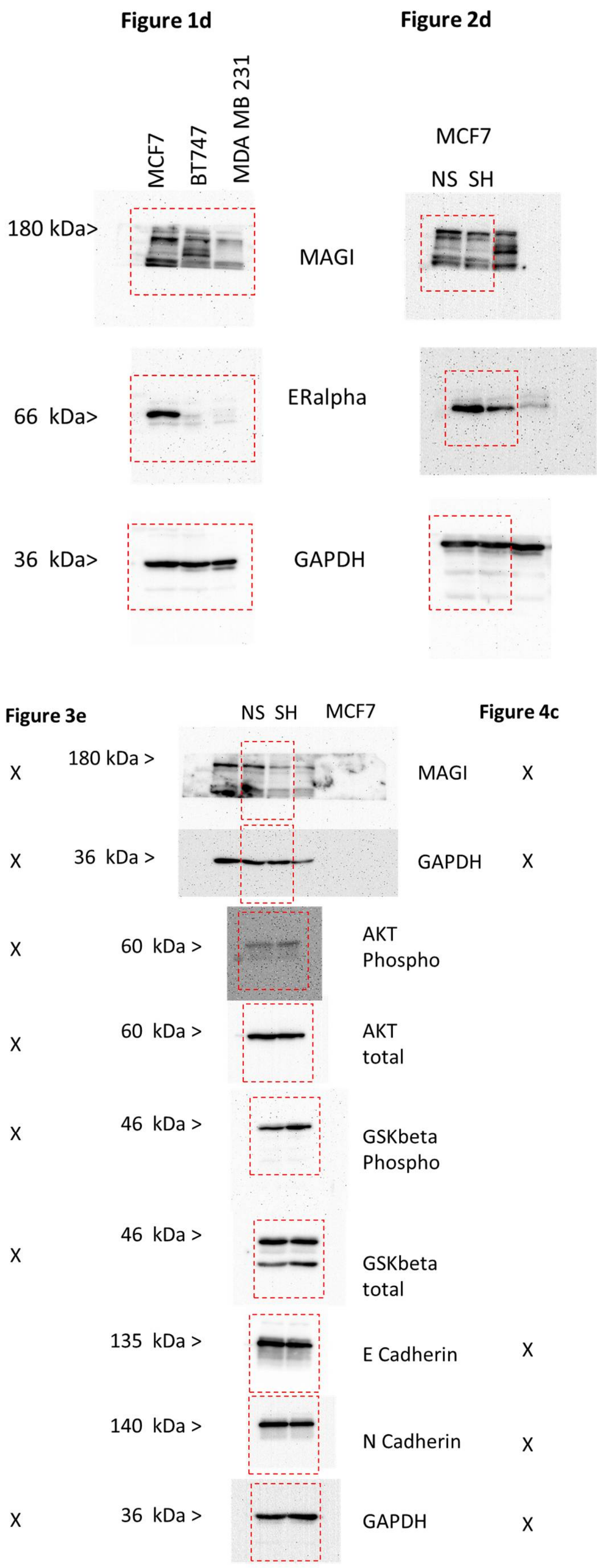
Figure 1d
Figure 2d
MCF7
BT747
MDA MB 231
MCF7
NS SH
180 kDa>
MAGI
66 kDa>
ERalpha
36 kDa>
GAPDH
Figure 3e
NS SH
MCF7
Figure 4c
X
180 kDa >
MAGI
X
X
36 kDa >
GAPDH
X
X
60 kDa >
AKT Phospho
X
60 kDa >
AKT total
X
46 kDa >
GSKbeta Phospho
X
46 kDa >
GSKbeta total
135 kDa >
E Cadherin
X
140 kDa >
N Cadherin
X
X
36 kDa >
GAPDH
X

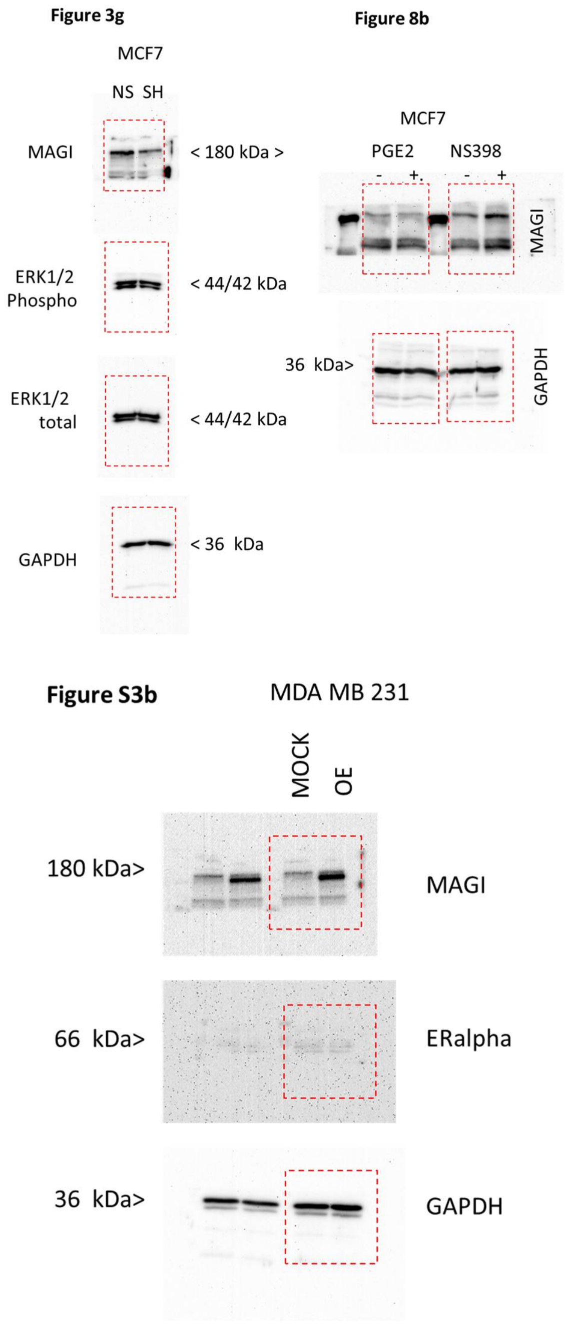

Figure S7. Original blot.

References

1. Haibe-Kains, B.; Desmedt, C.; Loi, S.; Culhane, A.C.; Bontempi, G.; Quackenbush, J.; Sotiriou, C.A three-gene model to robustly identify breast cancer molecular subtypes. *J. Natl. Cancer Inst.* **2012**, *104*, 311–325, doi:10.1093/jnci/djr545.
2. Sanjana, N.E.; Shalem, O.; Zhang, F. Improved vectors and genome-wide libraries for CRISPR screening. *Nat. Methods* **2014**, *11*, 783–784, doi:10.1038/nmeth.3047.

© 2020 by the authors. Licensee MDPI, Basel, Switzerland. This article is an open access article distributed under the terms and conditions of the Creative Commons Attribution (CC BY) license (http://creativecommons.org/licenses/by/4.0/).
